# Protein CoAlation and antioxidant function of coenzyme A in prokaryotic cells

**DOI:** 10.1042/BCJ20180043

**Published:** 2018-06-06

**Authors:** Yugo Tsuchiya, Alexander Zhyvoloup, Jovana Baković, Naam Thomas, Bess Yi Kun Yu, Sayoni Das, Christine Orengo, Clare Newell, John Ward, Giorgio Saladino, Federico Comitani, Francesco L. Gervasio, Oksana M. Malanchuk, Antonina I. Khoruzhenko, Valeriy Filonenko, Sew Yeu Peak-Chew, Mark Skehel, Ivan Gout

**Affiliations:** 1 Department of Structural and Molecular Biology, University College London, London WC1E 6BT, U.K.; 2 The Francis Crick Institute, London NW1 1AT, U.K.; 3 Department of Biochemical Engineering, University College London, London WC1E 6BT, U.K.; 4 Department of Chemistry, University College London, London WC1H 0AJ, U.K.; 5 Institute of Molecular Biology and Genetics, National Academy of Sciences of Ukraine, Kyiv 03680, Ukraine; 6 Biological Mass Spectrometry and Proteomics Cell Biology, MRC Laboratory of Molecular Biology, Cambridge CB2 0QH, U.K.

**Keywords:** coenzyme A, Gram-negative and -positive bacteria, post-translational modification, redox signaling

## Abstract

In all living organisms, coenzyme A (CoA) is an essential cofactor with a unique design allowing it to function as an acyl group carrier and a carbonyl-activating group in diverse biochemical reactions. It is synthesized in a highly conserved process in prokaryotes and eukaryotes that requires pantothenic acid (vitamin B5), cysteine and ATP. CoA and its thioester derivatives are involved in major metabolic pathways, allosteric interactions and the regulation of gene expression. A novel unconventional function of CoA in redox regulation has been recently discovered in mammalian cells and termed protein CoAlation. Here, we report for the first time that protein CoAlation occurs at a background level in exponentially growing bacteria and is strongly induced in response to oxidizing agents and metabolic stress. Over 12% of *Staphylococcus aureus* gene products were shown to be CoAlated in response to diamide-induced stress*. In vitro* CoAlation of *S. aureus* glyceraldehyde-3-phosphate dehydrogenase was found to inhibit its enzymatic activity and to protect the catalytic cysteine 151 from overoxidation by hydrogen peroxide. These findings suggest that in exponentially growing bacteria, CoA functions to generate metabolically active thioesters, while it also has the potential to act as a low-molecular-weight antioxidant in response to oxidative and metabolic stress.

## Introduction

Coenzyme A (CoA) is a ubiquitous and essential cofactor in all living cells, where it functions as a carbonyl-activating group and a carrier for activated acyl groups in numerous metabolic and catabolic processes. The biosynthesis of CoA in prokaryotes and eukaryotes is a conserved process that requires pantothenic acid (vitamin B5), cysteine and ATP. The presence of a thiol group in the CoA structure is at the core of its biochemical behavior. In cells, CoA forms a diverse range of thioester derivatives, such as Acetyl CoA, malonyl CoA and 3-hydroxy-3-methylglutaryl (HMG) CoA, which play central roles in many biochemical reactions in protein, carbohydrate and lipid metabolism [1–4]. These include the synthesis and oxidation of fatty acids, isoprenoid and cholesterol biosynthesis, amino acid metabolism, the Krebs cycle and the synthesis of peptidoglycans. In addition, CoA derivatives function as substrates for protein acylation (e.g. lysine acetylation succinylation, malonylation, propionylation and butyrylation), which has emerged as an important mechanism in the regulation of transcription, chromatin maintenance and cellular metabolism [5–7].

One aspect of CoA biochemistry that has not been well investigated is the role of this central metabolic coenzyme in thiol-disulfide exchange reactions and redox regulation. It has been reported that CoA undergoes copper-catalyzed air oxidation at a rate which is 4-fold slower than GSH (glutathione) and 720-fold less rapidly than cysteine, making it an appropriate protective thiol in all living cells [8]. As a thiol-containing molecule, CoA has been found to form CoA disulfides (CoASSCoA) and mixed disulfides with other low-molecular-weight (LMW) thiols (e.g, CoA-cysteine and CoA-glutathione, CoASSG) or cysteine residues in specific proteins. The CoASSG heterodimer has been isolated from bacteria, yeast, human myocardial tissue and parathyroid glands [9–11]. Potent vasoconstrictive and proliferative effects of CoASSG were observed in cultured vascular smooth muscle cells [12]. CoASSG was also shown to inhibit the activity of bacterial RNA polymerase [13].

Exposed protein thiols are the predominant targets of redox-linked regulation mediated by post-translational modifications, including oxidation, S-acylation, S-nitrosation, persulfhydration and S-thiolation [14,15]. When the cysteine thiol group is oxidized by reactive oxygen species (ROS) to an unstable sulfenic acid intermediate, it can react with nearby thiols leading to the formation of intra- and intermolecular disulfides or mixed disulfides with LMW thiols, such as cysteine, glutathione, bacillithiol and CoA. Formed disulfides are reversible regulatory events and function to protect unstable sulfenic acids against overoxidation to sulfinic and sulfonic acids which may alter irreversibly the structure, function and subcellular localization of modified proteins [16].

The formation of mixed disulfides between CoA and cysteines of specific proteins has been reported in several biochemical and crystallographic studies [17–21]. The CoA-modified forms of acetyl-CoA acetyltransferase and glutamate dehydrogenase were detected immunohistochemically in rat liver mitochondria [17,18]. In these studies, the activity and half-life of acetyl-CoA acetyltransferase were shown to be modified by covalent attachment of CoA. Furthermore, covalent modification of phenol sulfotransferase by CoA was shown to inhibit its activity in a dose- and time-dependent manner [19]. In *Klebsiella pneumoniae*, CoA binding to flavodoxin NifF was found to halt the N_2_ fixation by blocking electron transfer from pyruvate-flavodoxin oxidoreductase NifJ to nitrogenase NifH [20]. Covalent binding of CoA to the *Bacillus subtilis* organic peroxide sensor OhrR was reported, but the consequence of this modification on the transcription repressor activity of OhrR in oxidative stress response has not been examined [21]. Despite the existence of these sporadic studies, investigation into the extent of covalent protein modification by CoA and the mechanism of regulation in eukaryotes and prokaryotes has long been overdue. Recent studies from our laboratory revealed extensive covalent modification of cellular proteins by CoA in mammalian cells and tissues, which we termed protein CoAlation [22]. We showed that protein CoAlation is a reversible post-translational modification induced in mammalian cells by oxidizing agents and metabolic stress. To uncover protein CoAlation as a common post-translational modification and to reveal its role in redox regulation, we have developed a range of new research tools and methodologies, including: (a) unique anti-CoA mAbs which work efficiently in various immunological assays; (b) *in vitro* protein CoAlation and assay and (c) a reliable strategy for the identification of CoAlated proteins by LC–MS/MS.

In the present paper, we provide evidence that protein CoAlation occurs at a low level in Gram-negative and Gram-positive bacteria under normal growth conditions, but is strongly induced in response to oxidizing agents and metabolic stress. Approximately 12% of the predicted *Staphylococcus aureus* proteome was found to be CoAlated in diamide-treated bacteria. SaGAPDH (*S. aureus* glyceraldehyde-3-phosphate dehydrogenase), a key enzyme in glycolysis, was found to be readily CoAlated in *Escherichia coli* treated with hydrogen peroxide (H_2_O_2_), diamide and sodium hypochlorite (NaOCl). Furthermore, *in vitro* CoAlation of recombinant SaGAPDH prevented overoxidation and irreversible loss of its activity in the presence of exogenous H_2_O_2_. Altogether, our findings suggest that in bacteria, protein CoAlation is a widespread redox-regulated post-translational modification with a potential to protect critical reactive cysteines against irreversible overoxidation.

## Experimental

### Reagents and chemicals

All common chemicals were obtained from Sigma–Aldrich unless otherwise stated. The generation and characterization of the anti-CoA antibody (1F10) was described recently [23]. For Western blotting, anti-CoA antibody was diluted in Odyssey blocking buffer (0.17 µg/ml) containing 0.01% Tween 20. Secondary antibodies [Alexa Fluor 680 goat antimouse IgG H&L (Life Technologies)] were diluted in Odyssey blocking buffer (1 : 10 000) containing 0.02% sodium dodecyl sulfate (SDS).

### Bacterial species, growth conditions and treatments

Following bacterial species were used in the present study: *E. coli* SG13009 and DH5alpha, *Bacillus megaterium* NCTC10342 and *S. aureus* DSM11729. *B. megaterium* cells were cultured overnight in Nutrient Broth 3 (NB3) medium, while *E. coli* and *S. aureus* cells were grown in Luria Bertani (LB) medium. The overnight cultures were diluted 1 : 100 in the same media and incubated until the optical density of 0.7 at 600 nm (OD_600_). The samples of cells were then treated with or without oxidizing agents for 30 min at 37°C: hydrogen peroxide (10 and 100 mM), diamide (2 mM) and NaOCl (150 µM). To induce metabolic stress, bacterial cultures at OD_600_ of 0.7 were harvested by centrifugation and resuspended in M9 minimal medium supplemented with or without glucose as a source of carbohydrate.

### Cell lysis and protein extraction

Protein extracts from harvested bacteria were prepared in the following ways: (a) the pellet of harvested *E. coli*, *B. megaterium* and *S. aureus* was resuspended in buffer containing 100 mM Tris–HCl, pH 7.5, 100 mM NaCl, 100 mM NEM and a cocktail of protease inhibitors (Roche). SDS was added (1% final), and the homogenate was sonicated to reduce viscosity before centrifuging at 21 000 ***g*** for 10 min at RT. The supernatant was collected and analyzed by western blotting. (b) The pellet of harvested *S. aureus* was resuspended in buffer containing 100 mM Tris–HCl, pH 7.5, 100 mM NaCl, 100 mM NEM and a cocktail of protease inhibitors (Roche). To solubilize cell wall proteins, lysostaphin (22 U/ml) was added and the lysate was incubated at 37°C for 30 min. After the addition of SDS (1% final), the homogenate was sonicated to reduce viscosity before centrifuging at 21 000 ***g*** for 10 min at RT. The supernatant was collected for further analysis.

### Western blot analysis

Samples of bacterial extracts containing ∼30–40 µg of proteins were heated for 5 min at 99°C in SDS loading buffer with or without dithiothreitol (DTT, 100 mM final) and separated by SDS–polyacrylamide gel electrophoresis (PAGE) on 4–20% Mini-PROTEAN TGX Precast Gels (Bio-Rad Laboratories). Separated proteins were transferred from the gel to a low-fluorescence polyvinylidene fluoride membrane (Bio-Rad Laboratories), which was then blocked with Odyssey blocking buffer (LI-COR Biosciences). Primary anti-CoA antibody was diluted in Odyssey blocking buffer (0.17 µg/ml) and incubated with the membrane for 2 h at RT or overnight at 4°C. Immunoreactive protein bands were visualized using infrared dye-conjugated secondary antibodies and the Odyssey infrared imaging system (Odyssey Scanner CLx and Image Studio Lite software, LI-COR Biosciences).

### Expression and affinity purification of SaGAPDH

The full coding sequence of SaGAPDH was cloned into the pQE3/SaGAPDH expression plasmid with the N-terminal His-tag sequences as previously described [24]. Expression of His-tagged SaGAPDH was carried out in exponentially growing SG13009 cells in the presence of 1 mM isopropyl- β-d-thiogalactopyranoside (IPTG). After 3 h induction at 37°C, the cells were harvested and stored at −80°C. Affinity purification of His-tagged SaGAPDH was performed using Ni-NTA chromatography. Eluted preparations were examined by SDS–PAGE and stored at −80°C.

### GAPDH enzymatic assay

Recombinant SaGAPDH activity was determined by measuring the absorbance change at 340 nm and 25°C resulting from the production of NADH. The reaction was carried out in a 150 µl assay mixture containing 20 mM Tris–HCl (pH 8.7), 0.36 µM SaGAPDH, 1.25 mM NAD^+^, 1.25 mM ethylenediaminetetraacetic acid and 15 mM sodium arsenate. The reaction was started by the addition of 0.25 mM glyceraldehyde 3-phosphate. Initial reaction rates were calculated as described recently [25], by determining the slope in the linear part of the curve during the first 80 s of the reaction (GraphPad, linear regression function). The percentage of SaGAPDH activity was calculated as: Rate of inactivated/Rate of untreated × 100%. The results are presented as mean ± SEM from at least three separate experiments.

For the inactivation experiments, SaGAPDH was preincubated with 1 µM, 10 µM, 100 µM and 10 mM H_2_O_2_ for 10 min or with 10 mM CoASSCoA for 30 min. About 2 µl of the mixture was then added to the assay mixture and the remaining activity was measured as described. To reduce it, the enzyme was incubated with 10 mM DTT for 15 min. After treatments, excess H_2_O_2_, CoASSCoA and DTT were removed using Micro Biospin 6 columns (Bio-Rad).

### Purification and activity assay of Nudix 7 hydrolase

Recombinant His-Nudix 7 hydrolase was expressed in bacteria and purified by Ni-NTA affinity chromatography. His-Nudix 7 (1.7 µg) was incubated in a total volume of 100 µl containing 50 mM (NH_4_)HCO and 0.2 mM CoASSG at 37°C for 20 min with or without 5 mM MgCl_2_. Reaction products and substrates were analyzed by HPLC as described, except that elution was monitored at 205 nm [26].

### Preparation and enrichment of CoAlated peptides from diamide-treated *S. aureus* for MS analysis

The pellet of diamide-treated *S. aureus* (2 mM for 30 min) was resuspended in buffer containing 100 mM Tris–HCl, pH 7.5, 100 mM NaCl, 100 mM NEM and a cocktail of protease inhibitors (Roche). The lysate was incubated with lysostaphin (20 U/ml) at 37°C for 30 min to solubilize cell wall proteins. SDS was then added (1% final), and the homogenate was sonicated to reduce viscosity before centrifuging at 21 000 ***g*** for 10 min at RT. Proteins in the supernatant were precipitated with 90% methanol. The protein pellet was resuspended in 50 mM (NH_4_)HCO_3_ (pH 7.8) supplemented with 6.4 mM iodoacetamide (IAM) and digested with endoproteinases Lys C and trypsin (sequencing grade, Promega). After heat inactivation (99°C, 10 min) of digestive enzymes, CoAlated peptides were immunoprecipitated with anti-CoA antibody cross-linked to Protein G Sepharose. Trypsin digested and immunoprecipitated peptide mixtures were dried down completely in a SpeedVac and resolubilized in 20 µl of 50 mM ammonium bicarbonate (Ambic). After mixing for 2 min, 2.3 µl of 50 mM MgCl_2_ was added followed by 1 µl of Nudix 7 phosphatase. The solution was incubated at 37°C for 20 min then acidified, desalted with a C_18_ Stage tip that contained 1.5 µl of Poros R3 resin and partially dried in a SpeedVac. Modified peptides were further enriched using Phos-Select IMAC resin (Sigma). Desalted peptides were resuspended in 100 µl of 30% MeCN, 0.25 M acetic acid (loading solution) and 30 µl of IMAC beads, previously equilibrated with the loading solution was added. After 45 min incubation at room temperature, beads were washed four times with loading solution and CoAlated peptides were eluted twice with 500 mM imidazole (pH 7.6) and once with 30% MeCN/500 mM imidazole (pH 7.6). CoAlated peptides were acidified, dried and desalted with a C18 Stage tip that contained 1.5 µl of Poros R3 resin. This solution was then partially dried down using a SpeedVac and was ready for mass spectrometry analysis.

### *In vitro* CoAlation of SaGAPDH

About 100 µg of affinity-purified SaGAPDH was CoAlated in buffer containing 50 mM Tris-HCl, pH 7.5 and 250 µM CoASSCoA for 30 min. Excess of unbound CoASSCoA was removed using Micro Biospin 6 columns (Bio-Rad).

### Preparation and enrichment of CoAlated peptides from *in vitro* CoAlated SaGAPDH

Solution samples of *in vitro* CoAlated SaGAPDH (2 µg) in 50 mM ammonium bicarbonate (NH_4_CO_3_) and 8 mM iodoacetamide were digested with endoproteinases Lys C and elastase (Promega, U.K.). To the peptide mixture, 3.1 µl of 50 mM MgCl_2_ was added followed by 1.35 µl of Nudix-7 phosphatase and the mixture was incubated at 37°C for 20 min. The peptide mixture was then acidified, desalted on a C18 Stage tip (3M Empore) containing 0.7 µl of Poros R3 resin (Applied Biosystems, U.K.) and partially dried in a SpeedVac.

CoAlated peptides were enriched using Phos-Select IMAC resin (Sigma, U.K.). Desalted peptides were resuspended in 100 µl of 30% (v/v) acetonitrile (MeCN), 0.25 M acetic acid (loading solution) and 10 µl of IMAC resin, previously equilibrated with the loading solution was added. After 45 min incubation at room temperature, the resin was washed four times with loading solution and the CoAlated peptides were eluted with 500 mM imidazole (pH 7.6) followed by 30% (v/v) MeCN/500 mM imidazole (pH 7.6). CoAlated peptides were acidified, dried and desalted with a C18 Stage tip containing 0.5 µl of Poros R3 resin (Applied Biosystems, U.K.). The solution was then partially dried down using a SpeedVac prior to analysis by mass spectrometry.

### Mass spectrometry and data acquisition

Mass spectrometry data acquisition liquid chromatography was performed on a fully automated Ultimate U3000 Nano LC System (Dionex) fitted with a 100 µm × 2 cm PepMap100 C_18_ Nano-Trap column and a 75 μm × 25 cm reverse-phase PepMap100 C_18_ Nano-Trap column (Dionex). Peptides were separated using an acetonitrile gradient and sprayed directly via a nano-flow electrospray ionization source into the mass spectrometer (Orbitrap Velos, Thermo Scientific). The mass spectrometer was operated in a standard data-dependent mode, performed survey full scan (*m*/*z* = 350–1600) in the Orbitrap analyzer, with a resolution of 60 000 at *m*/*z* = 400, followed by MS/MS acquisitions of the 20 most intense ions in the LTQ ion trap. Maximum FTMS scan accumulation times were set at 250 ms and maximum ion trap MSn scan accumulation times were set at 200 ms. The Orbitrap measurements were internally calibrated using the lock mass of polydimethylcyclosiloxane at *m*/*z* 445.120025. Dynamic exclusion was set for 30 s with exclusion list of 500.

### Data processing

LC–MS/MS raw data files were processed as standard samples using MaxQuant version 1.5.2.8, which incorporates the Andromeda search [27]. MaxQuant processed data were searched against a Uniprot — *S. aureus* (November 2015) database. Carbamidomethyl cysteine, acetyl N-terminal, *N*-ethylmaleimide cysteine, oxidation of methionines, CoAlation of cysteine with *λ* mass 338, 356 and 765 were set as variable modifications. For all data sets, the default parameters in MaxQuant were used, except MS/MS tolerance which was set at 0.6 Da and the second peptide ID was unselected.

Using MQ viewer, CoA_356 peptides were first visually checked. Those matched MS/MS spectra that did not have continuous 4 *y* or *b* ion series were checked manually.

### Functional characterization of identified proteins

Gene ontology (GO) [28] terms describing the function(s) of the identified proteins were either extracted from UniProtKB (UniProt Release June 2017) or predicted using a protein domain-based function prediction pipeline [29,30]. The functions of the proteins were then classified into major functional categories and protein classes were based on the inferred GO terms.

### Molecular dynamics simulations

A high-resolution (1.7 Å) X-ray crystal structure of glyceraldehyde 3-phosphate dehydrogenase (GAPDH) complexed with NAD^+^, from *S. aureus*, was obtained from the Protein DataBank (PDBID: 3LVF). Missing residues were modeled with MODELER [31]. The molecular dynamics (MD) simulations were performed using the code GROMACS 4 [32]. To enhance the sampling, we used the Metadynamics algorithm as implemented the PLUMED plug-in [33,34]. The protein was described by the Amber99SB*-ILDN force field which includes the dihedral corrections of Best and Hummer, while CoA was parametrized with the general Amber force field (GAFF) and RESP charges derived from *ab initio* calculations at the Hartree–Fock level of theory [35,36]. The system was solvated with ∼19 000 tip3p water molecules and enclosed in a dodecahedron box with periodic boundary conditions for a total of more than 60 000 atoms. The van der Waals interactions were smoothly shifted to zero between 0.8 and 1.0 nm; the long-range electrostatic interactions were calculated by the particle mesh Ewald algorithm, with mesh spaced 0.12 nm, combined with a switch function for the direct space between 0.8 and 1.0 nm for better energy conservation [37,38]. Following an initial conjugate gradient optimization to relax the structure and remove possible atomic clashes, a brief NPT equilibration was run with a Berendsen thermostat and target pressure of 1 bar. The system evolved in the canonical ensemble with a time step of 2 fs and was coupled with a velocity-rescale thermostat to maintain the temperature at 300 K.

### Statistical analysis

Where appropriate, values are given as means ± SEM. Graphs were produced and statistics were calculated using GraphPad Prism (version 6.07 for Windows, GraphPad Software, La Jolla, CA, U.S.A.; www.graphpad.com).

## Results

### Oxidizing agents induce strong protein CoAlation in bacteria

Bacteria employ a diverse range of molecular mechanisms to cope with ROS/reactive nitrogen species and to repair the resulting damage [39]. These include the production of antioxidant enzymes (superoxide dismutase, catalases and peroxiredoxins) and LMW thiols (glutathione, bacillithiol and mycothiol). While significant progress has been made to understand the antioxidant function of glutathione and to some extent bacillithiol and mycothiol, the role of the small thiol CoA in redox regulation in bacteria remains to be elucidated. This was mainly due to the lack of specific antibodies which can recognize CoA in various immunological assays and a reliable mass spectrometry-based protocol for identifying CoA-modified peptides in CoAlated proteins. The developed research tools and methodologies and the identification of extensive protein CoAlation in mammalian cells induced by oxidizing agents and metabolic stress prompted us to investigate the magnitude and relevance of this post-translational modification in bacteria [22]. To do so, we used both Gram-negative (*E. coli*) and Gram-positive (*S. aureus* and *B. megaterium*) bacteria, which have different expression profiles of LMW thiols. In Gram-negative bacteria and eukaryotes, glutathione is the major LMW thiol and antioxidant; however, it is absent in most Gram-positive bacteria. Instead, the differential expression of bacillithiol and mycothiol has been reported in different species of Gram-positive bacteria, where they function as a thiol redox buffer in the detoxification of ROS, toxins and antibiotics [40]. In contrast, CoA is a ubiquitous and highly expressed LMW thiol in all living cells, whose function has been mainly associated with the regulation of cellular metabolism and gene expression.

**Figure 1.**
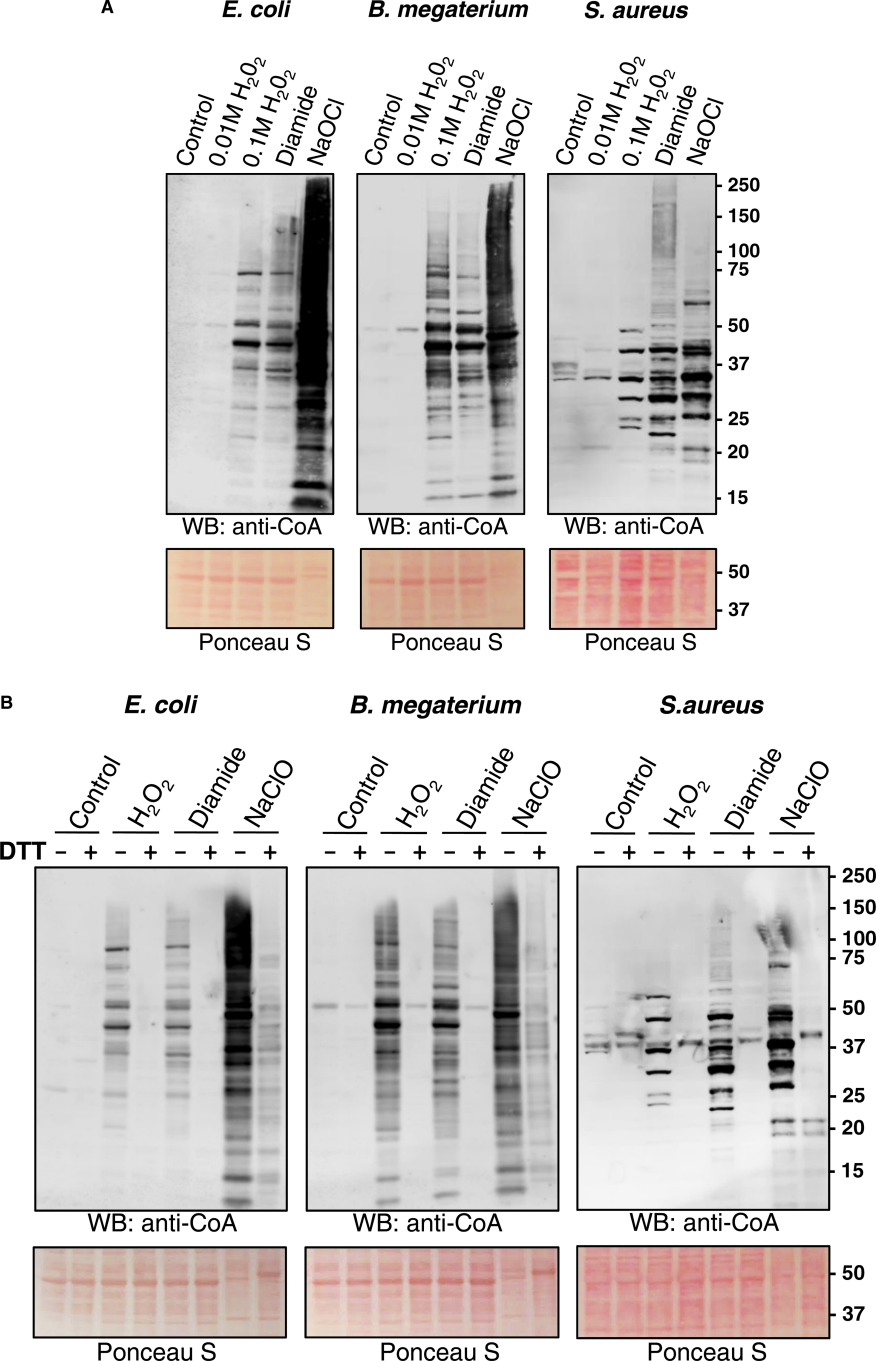
Induction of protein CoAlation in bacteria by a panel of oxidizing agents. Protein CoAlation in Gram-positive and Gram-negative bacteria is induced by different oxidizing agents (**A**) in a DTT-sensitive manner (**B**). *E. coli*, *B. megaterium* and *S. aureus* were grown to mid-log phase in rich medium at 37°C and then treated for 30 min with and without 10 mM or 100 mM H_2_O_2_, 2 mM diamide and 100 µM NaOCl. Cells were lysed as described in Experimental procedures and protein CoAlation examined by anti-CoA immunoblot. DTT (200 mM final) was added to protein extracts before SDS–PAGE analysis to demonstrate that the protein-CoA binding involves a reversible disulfide bond formation.

Initially, we examined the effect of H_2_O_2_ on protein CoAlation in *E. coli*, *B. megaterium* and *S. aureus*. Here, bacteria were grown to mid-log phase (OD_600_ = 0.7) in rich medium (LB medium for *E. coli* and *S. aureus*; NB3 medium for *B. megaterium*) at 37°C and then treated with and without 10 or 100 mM H_2_O_2_. After 30 min, cells were collected and bacterial protein extracts were prepared as described in the Experimental procedures. Separation of protein extracts under non-reducing conditions and Western blot analysis with anti-CoA antibody 1F10 revealed a weak immunoreactive signal in control samples and cells treated with 10 mM H_2_O_2_ (Figure 1A). However, exposure of cells to 100 mM H_2_O_2_ induced readily detectable protein CoAlation in all three bacterial species. These data indicate that bacteria can cope efficiently with oxidative stress induced by 10 mM H_2_O_2_, without engaging CoA in the antioxidant response. We also observed extensive protein CoAlation in cells treated with the disulfide stress inducer diamide at a concentration of 2 mM. Notably, the pattern of CoA-modified proteins in cells treated with H_2_O_2_ and diamide was similar except for several differentially CoAlated proteins (Figure 1A). Hypochlorous acid (HOCl) is produced by neutrophils to kill engulfed bacteria and is commonly used as an antimicrobial disinfectant [41]. The bactericidal effect of HOCl is associated with the production of various ROS. LMW thiols, such as GSH, protect bacteria from HOCl by direct interaction and the formation of less harmful substances. The treatment of bacteria with NaOCl was shown to induce the formation of mixed disulfides between LMW thiols and proteins, mediated by the disulfide exchange mechanism [25,42]. The treatment of exponentially growing bacteria with 100 µM NaOCl showed the strongest induction of protein CoAlation, when compared with control, H_2_O_2_ or diamide. The pattern of protein CoAlation differed significantly from that of H_2_O_2_- or diamide-treated cells. Ponceau staining of protein blots revealed that the treatment of cells with 100 µM NaOCl caused a significant change in the pattern of separated proteins, when compared with control and H_2_O_2_- or diamide-treated cells (Figure 1A).

To demonstrate that the protein-CoA binding involves a reversible disulfide bond formation, the disulfide reducing agent DTT was added to protein extracts before SDS–PAGE analysis. As shown in Figure 1B, the presence of 200 mM DTT in the sample buffer efficiently abrogated immunoreactive signal in H_2_O_2_- and diamide-treated cells. In case of hypochlorite stress, the reduction of protein-CoA disulfide bonds was not complete and possibly required a higher DTT concentration in the sample buffer.

**Figure 2.**
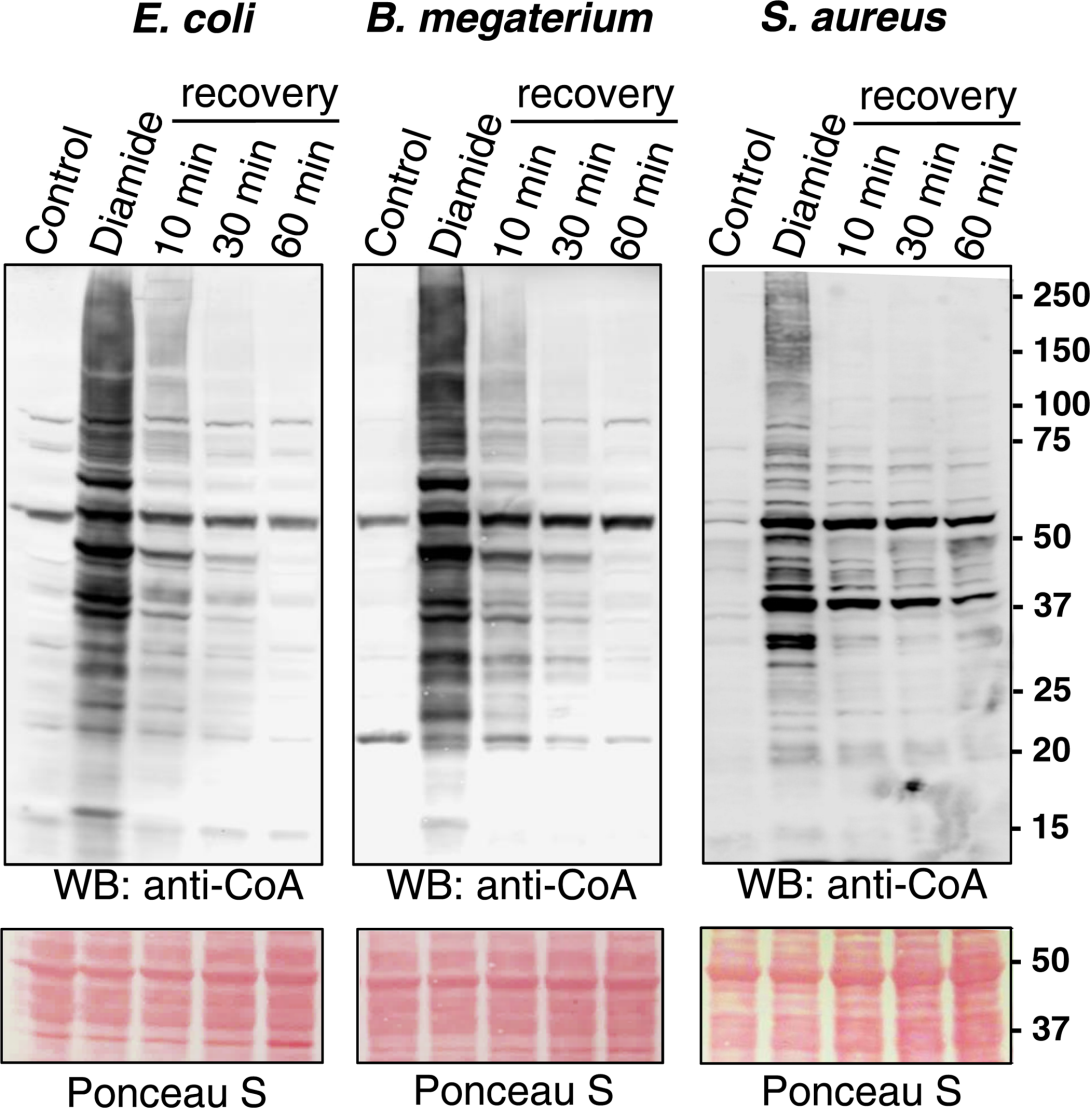
Diamide-induced protein CoAlation in *E. coli, B. megaterium* and *S. aureus* is a reversible post-translational modification. Exponentially growing bacteria were treated with 2 mM diamide for 30 min. The medium was then replaced with fresh media without the oxidant and cells were incubated for the indicated times. Protein CoAlation was examined by anti-CoA immunoblot.

To find out whether protein CoAlation is a reversible post-translational modification, exponentially growing *E. coli*, *B. megaterium* and *S. aureus* were treated with 2 mM diamide for 30 min. Bacteria were harvested by centrifugation and then incubated in fresh LB or NB3 media for various periods of time to recover from the oxidative stress. As shown in Figure 2, diamide-induced protein CoAlation in *E. coli* was reversed to the level of untreated cells in a time-dependent manner. The reversibility of protein CoAlation in *B. megaterium* and *S. aureus* also occurred in a time-dependent manner, but did not reach baseline levels within 60 min.

### Induction of protein CoAlation by glucose deprivation

Bacteria have evolved elaborate strategies that enable them to adapt to challenging growth environments. When the nutrient supply becomes limiting, bacteria employ general starvation-response mechanisms, such as the stringent response and carbon catabolite repression, which are associated with ROS production and oxidative stress [43]. We were interested to examine whether under nutrient deprivation CoA is also used for S-thiolation of redox-sensitive cysteine residues, resulting in the formation of mixed disulfides with proteins. Taking into account that the examined bacterial species use carbon catabolism for energy generation, we employed the model of glucose starvation.

**Figure 3.**
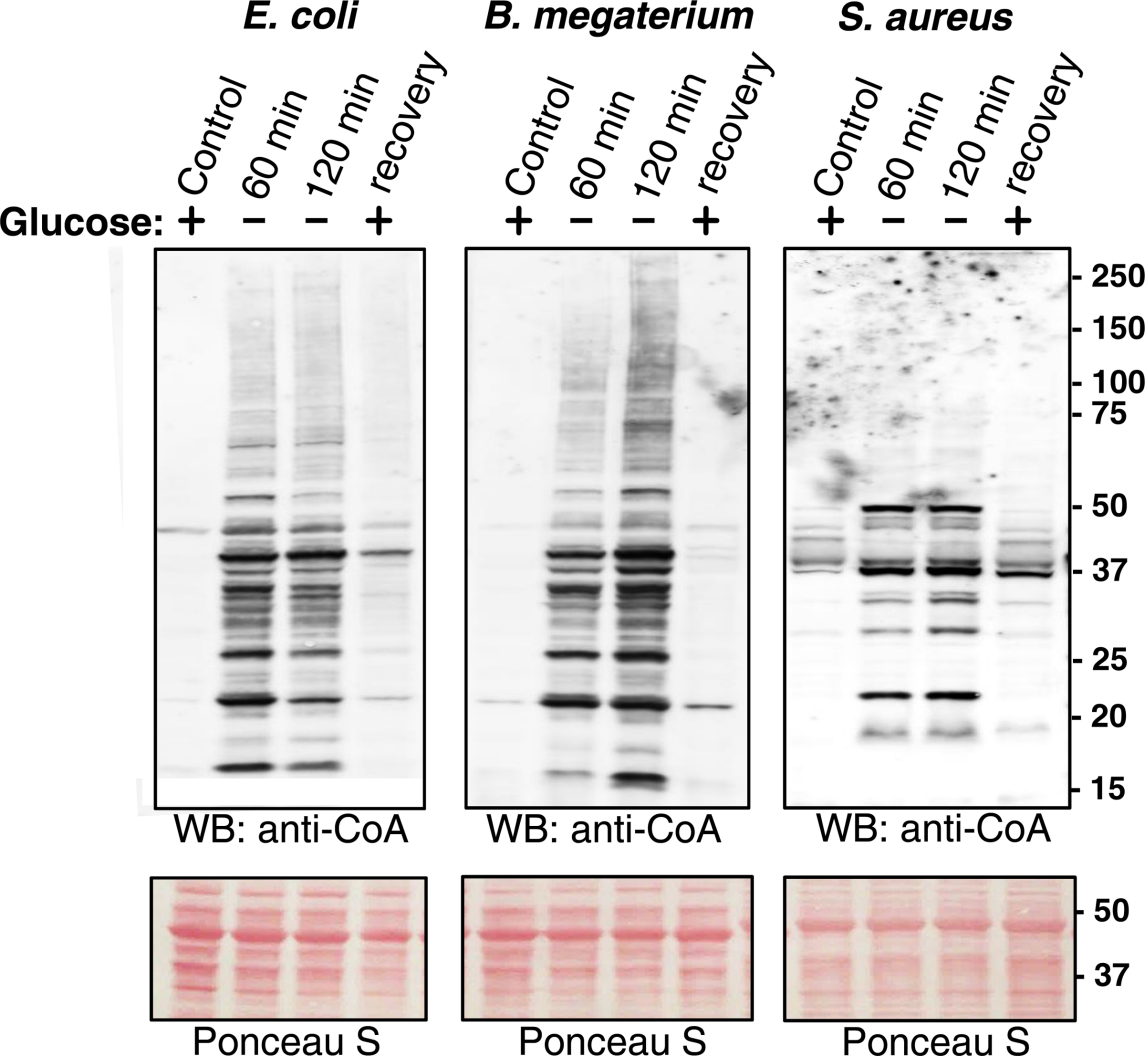
Induction of protein CoAlation by metabolic stress. Glucose depravation induces protein CoAlation in *E. coli*, *B. megaterium* and *S. aureus*, which is reversed by the re-addition of glucose. *E. coli*, *B. megaterium* and *S. aureus* were grown to mid-log phase in rich medium at 37°C and then transferred and cultured in the medium lacking glucose or any other source of carbohydrates for the indicated times. The cultures of glucose-starved bacteria were then supplemented with 20 mM glucose and incubated at 37°C for 30 min. Protein CoAlation in total protein extracts was examined by anti-CoA immunoblot.

In the present study, all three types of bacteria were cultured in nutrient-rich medium until OD_600_ = 0.7 and then transferred in the medium lacking glucose or any other source of carbohydrates. As shown in Figure 3, protein CoAlation was at a very low level in bacteria cultured in nutrient-rich medium, but strongly induced under the condition of glucose starvation for 60 and 120 min. The pattern of CoAlated proteins in glucose-starved *E. coli* and *B. megaterium* was similar, but differed significantly from that in *S. aureus*. Comparing the pattern of CoA-modified proteins induced by glucose starvation and the treatment with oxidizing agents revealed little similarity in all three types of examined bacteria, indicating the involvement of different redox-sensing and responding strategies.

We then examined whether protein CoAlation induced by glucose starvation can be reversed with the re-addition of glucose to starved bacterial cultures. The results presented in Figure 3 clearly indicate that supplementing cultures of glucose-starved bacteria with glucose for 30 min resulted in near complete deCoAlation of CoA-modified proteins.

### Mass spectrometry-based identification of CoAlated proteins in diamide-treated *S. aureus*

**Figure 4.**
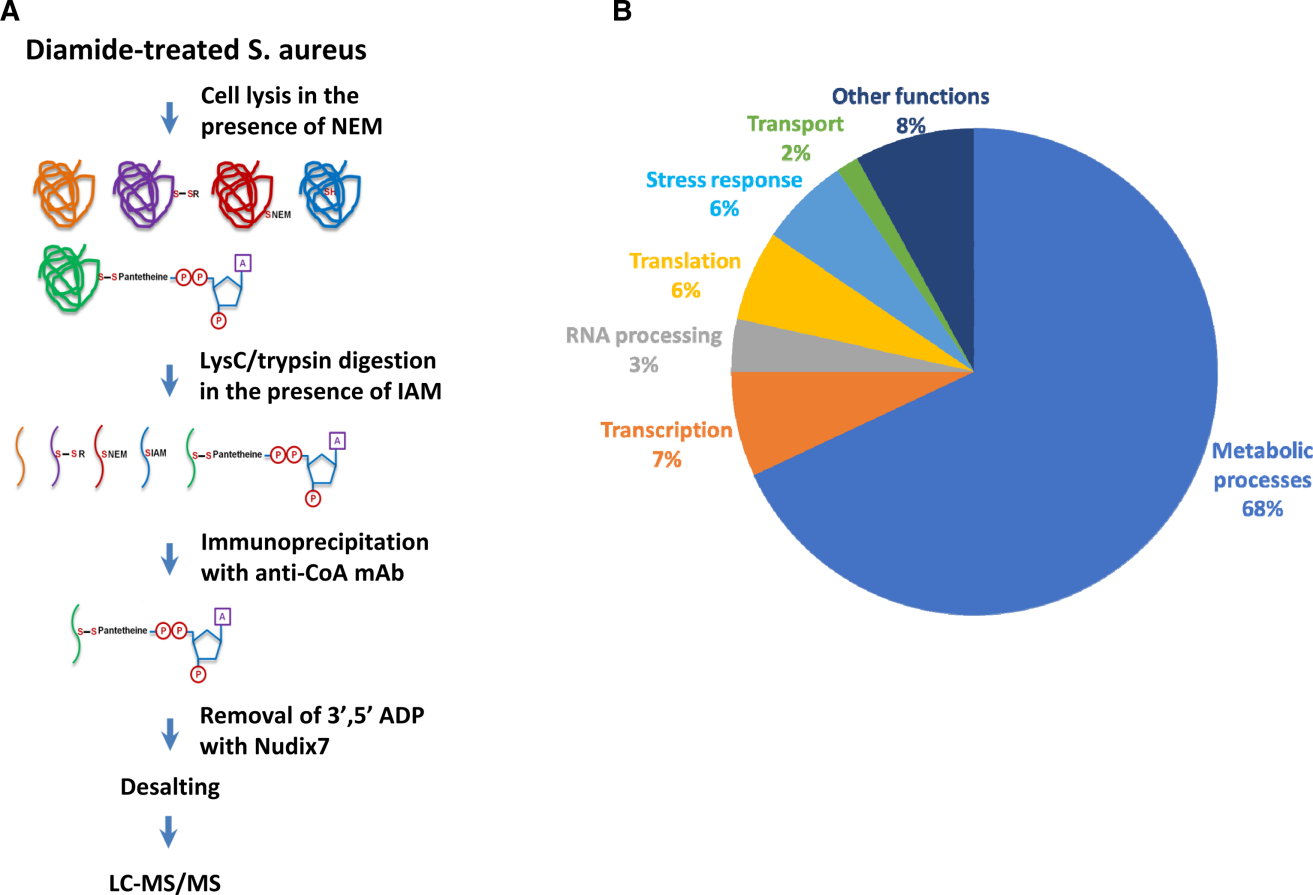
Development of methodology and the identification of CoAlated proteins. (**A**) Strategy for the identification of CoA-modified proteins *S. aureus* in response to diamide. (**B**) Pie chart showing the major functional categories of the proteins that were identified to be CoAlated in diamide-treated *S. aureus*.

Extensive protein CoAlation which we observed in Gram-negative and Gram-positive bacteria in response to oxidative and metabolic stress encouraged us to identify CoA-modified proteins using the developed methodology [22]. Our efforts were focused on determining the identity of CoAlated proteins in *S. aureus* under diamide-induced disulfide stress. Exponentially growing *S. aureus* were treated with 2 mM diamide for 30 min and protein extracts were prepared as described in Experimental procedures. In brief, to prevent *in vitro* modification of protein thiols by free CoA, 25 mM NEM was added to the lysis buffer. Extracted proteins were digested with Lys C/trypsin in the presence of IAM and CoAlated peptides were immunoprecipitated with anti-CoA antibody. Then, immune complexes were incubated with Nudix 7 hydrolase to remove the ADP moiety of CoA and to produce a distinctive MS/MS fragmentation signature of Cys + 356, corresponding to covalently attached 4PP (4′-phosphopantetheine). Representative MS/MS spectrum of a cysteine-containing peptide from SaGAPDH is shown in Figure 4A. In total, the LC–MS/MS analysis revealed the identity of 440 CoAlated cysteine-containing peptides which correspond to 356 proteins in the *S. aureus* proteome (Table 1). Bioinformatic pathway analysis revealed that a large number of CoAlated proteins are involved in major metabolic pathways, regulation of transcription, protein synthesis and stress response (Figure 4B). Among identified proteins, we found those which use CoA as the covalent intermediate in catalytic reactions or function as CoA-regulated proteins. These include succinate-CoA ligase, acyl-CoA ligase, HMG-CoA synthase, an acetyl-CoA carboxylase and acyl-CoA dehydrogenase.

**Table 1:** Proteomic identification of CoAlated proteins in *S. aureus* treated with diamide. CoAlated peptides identified by MS/MS analysis and corresponding proteins are shown. Perspective CoA-modified cysteine residues within the identified peptides are marked by asterisks.

Gene name	Protein name	MW (kDa)	Sequence	Score
HMPREF0769_11633	Resolvase, N-terminal domain protein	21.845	LNAYGC*EK	72.531
MW2460	MW2460 protein	63.757	LC*EDVAVYNHQIEK	97.291
ybaK	Cys-tRNA(Pro)/Cys-tRNA(Cys) deacylase	17.89	GGC*SPVGMK	41.689
pyrE	Orotate phosphoribosyltransferase	22.057	SPIYC*DNR	105.25
binL	BinL protein	22.491	LNTHGC*EK	98.156
SAOUHSC_02733	Membrane protein, putative	67.572	NVNVC*TIPFK	116.98
SAUSA300_1090	Pseudouridine synthase	34.634	DYTLVEC*QLETGR	88.803
SAKOR_01553	Uncharacterized protein	50.22	LIEESPC*AALTEER	106.87
pyrG	Cytidine 5′-triphosphate synthase	59.991	ESVIEC*R	131.12
pyrG	Cytidine 5′-triphosphate synthase	59.991	IALFC*DINK	153.34
pyrG	Cytidine 5′-triphosphate synthase	59.991	LGLYPC*SIK	86.114
yisK	Fumarylacetoacetate hydrolase family protein	33.113	SLTGGC*PMGPYIVTK	108.45
deoC2	Deoxyribose-phosphate aldolase 2	23.341	SVC*VNPTHVK	99.941
polC	DNA polymerase III PolC-type	162.69	NC*GFDIDK	94.767
AYM28_04315	Phage protein	10.633	ENYFC*DR	58.132
V070_02571	Uncharacterized protein	13.806	SC*VEVAR	83.647
pflB	Formate acetyltransferase	84.861	AAC*EAYGYELDEETEK	166.57
pflB	Formate acetyltransferase	84.861	IPYDC*C*K	101.46
rpmF	50S ribosomal protein L32	6.48	NC*GSYNGEEVAAK	159.4
purB	Adenylosuccinate lyase	49.603	EELDEC*FDPK	109.15
pheT	Phenylalanine–tRNA ligase β subunit	88.901	AC*YLLQTYANGK	110.22
SAZ172_1072	Uncharacterized protein	8.7688	NAGKFEETPC*EFVDGSKGVR	236.03
ddl	d-alanine–d-alanine ligase	40.23	ATDC*SGLVR	136.81
ddl	d-alanine–d-alanine ligase	40.23	C*NNEAELK	100.09
HMPREF0769_11996	Response regulator receiver domain protein	27.021	KDGIDVC*K	104.94
gapA2	Glyceraldehyde-3-phosphate dehydrogenase 2	36.979	SC*NESIIPTSTGAAK	122.83
N/A	Truncated catalase-like protein	38.88	GVGIENIC*PFSR	173.19
mvaD	Mevalonate diphosphate decarboxylase	36.831	EAGYPC*YFTMDAGPNVK	116.79
rpoC	DNA-directed RNA polymerase subunit β′	135.41	C*GVEVTK	114.89
rpoC	DNA-directed RNA polymerase subunit β′	135.41	DGLFC*ER	87.806
rpoC	DNA-directed RNA polymerase subunit β′	135.41	DWEC*.SC*.GK	121.61
rpoC	DNA-directed RNA polymerase subunit β′	135.41	MYQC*GLPK	123.86
gluD	NAD-specific glutamate dehydrogenase	45.76	C*GIVNLPYGGGK	135.81
gluD	NAD-specific glutamate dehydrogenase	45.76	GGIVC*DPR	110.53
SAKOR_01005	Phosphoenolpyruvate–protein phosphotransferase	63.276	LC*LAQQDIFR	135.02
SAKOR_02109	Uncharacterized protein	12.497	TAETNYFWLNC*GYNR	141.34
HMPREF0776_2410	ABC transporter, ATP-binding protein	13.659	FTEGNC*YGLIGANGAGK	128.85
SAOUHSC_00756	Uncharacterized protein	41.797	IAELC*HK	96.034
yloU	General stress protein, Gls24 family	13.345	AVEC*YGIVGMASR	161.48
rap	50S ribosomal protein L2	30.026	MILSTC*R	128.6
cmk	Cytidylate kinase	24.595	GQC*VILDNEDVTDFLR	91.622
sarS	HTH-type transcriptional regulator SarS	29.889	KIVSDLC*YK	205.53
SAR2771	UPF0176 protein SAR2771	36.938	DWFDGKPC*ER	108.32
SAR2771	UPF0176 protein SAR2771	36.938	VVTYC*TGGIR	139.78
SAR2771	UPF0176 protein SAR2771	36.938	YINCANPEC*NK	111.07
SAR2771	UPF0176 protein SAR2771	36.938	YLGAC*SYDCAK	92.151
SAR2771	UPF0176 protein SAR2771	36.938	YLGACSYDC*AK	104.7
SAR2771	UPF0176 protein SAR2771	36.938	YTTIDDPEQFAQDHLAFC*K	97.823
HMPREF0769_10405	Metallo-β-lactamase domain protein	29.57	EVLLC*DTDK	190.66
ctsR	Transcriptional regulator CtsR	17.841	FDC*VPSQLNYVIK	189.52
rplN	50S ribosomal protein L14	13.135	FDENAC*VIIR	96.464
rplN	50S ribosomal protein L14	13.135	TANIGDVIVC*TVK	196.01
RK97_03585	Nitrogen fixation protein NifU	16.631	C*ATLAWK	95.81
RK97_03585	Nitrogen fixation protein NifU	16.631	GVLDNGSMTVDMNNPTC*GDR	109.28
proC	Pyrroline-5-carboxylate reductase	29.825	QQLEC*QNPVAR	108.23
aspS	Aspartate–tRNA ligase	55.836	C*FRDEDLR	94.767
infB	Translation initiation factor IF-2	31.699	VGTIAGC*YVTEGK	132.15
pyrB	Aspartate carbamoyltransferase	33.257	GESLYDTC*K	130.69
tsaD	tRNA N6-adenosine threonylcarbamoyltransferase	37.069	QSLADQC*K	72.289
mshB	Bacillithiol biosynthesis deacetylase BshB2	24.894	ERELEEAC*K	158.76
SAOUHSC_00547	Uncharacterized protein	58.418	QC*QDISQYIENK	94.309
murI	Glutamate racemase	29.702	C*PYGPRPGEQVK	103.13
clpC	ATP-dependent Clp protease	91.141	GELQC*IGATTLDEYRK	190.1
SAV0414	Uncharacterized protein	83.287	C*AIVTDLDEQAIPSEHR	77.668
SAV0414	Uncharacterized protein	83.287	IVEFEAC*R	149.94
SAV0414	Uncharacterized protein	83.287	SNLNFC*INENYDK	231.38
murE	UDP-*N*-acetylmuramoyl-l-alanyl-d-glutamate–l-lysine ligase	54.104	FC*QNVADQGCK	82.515
SAOUHSC_02158	Uncharacterized protein	48.119	IAFSC*VEK	131.42
ribH	6,7-Dimethyl-8-ribityllumazine synthase	16.41	GATSHYDYVC*NEVAK	54.094
nadE	NH(3)-dependent NAD(+) synthetase	30.682	EEGIDC*TFIAVK	139.77
glyA	Serine hydroxymethyltransferase	45.172	EAEETLDSVGITC*NK	114.55
glyA	Serine hydroxymethyltransferase	45.172	GGMILC*K	85.212
AYM28_08455	Integral membrane protein	36.972	YSYIC*EK	125.75
ble	Bleomycin resistance protein	14.922	SIGFYC*DK	117.25
SAOUHSC_01732	Uncharacterized protein	12.768	KEGQGC*ISLK	92.247
typA	GTP-binding protein TypA	37.865	EIDGVMC*EPFER	191.89
secA	Protein translocase subunit SecA	11.664	NDDC*.PC*.GSGK	70.68
vraX	Protein VraX	6.5003	C*DDSFSDTEIFK	128.06
AYM28_07645	YqiW-like protein	16.014	DAFDENC*K	129
AYM28_05325	UPF0738 protein AYM22_05325	13.529	IKDDILYC*YTEDSIK	108.28
dltA	d-alanine–poly(phosphoribitol) ligase subunit 1	54.67	AGC*GYVPVDTSIPEDR	167.93
SAOUHSC_02613	Uncharacterized protein	25.225	AVC*GFSK	90.601
SAOUHSC_01744	Uncharacterized protein	85.61	VLNDQC*PTSVK	153.41
AYM28_02495	GIY-YIG catalytic domain protein	9.1542	C*SDGSLYTGYAK	114.31
tagF_3	CDP-glycerol–poly(glycerophosphate) glycerophosphotransferase	66.074	IDGNQFVC*R	111.5
mqo1	Probable malate : quinone oxidoreductase 1	54.785	C*TNQEVIDR	135.04
mqo1	Probable malate : quinone oxidoreductase 1	54.785	DGTVDC*SK	147.3
N/A	Putative uncharacterized protein	7.9711	GEC*DDKWEGLYSK	104.76
gnd	6-Phosphogluconate dehydrogenase	51.802	DGASC*VTYIGPNGAGHYVK	98.76
gnd	6-Phosphogluconate dehydrogenase	51.802	IC*SYAQGFAQMR	155.33
alr1	Alanine racemase 1	42.823	VC*MDQTIVK	114.72
SAOUHSC_00696	Uncharacterized protein	34.777	C*GIILPSK	110.41
ychF	Ribosome-binding ATPase YchF	13.516	EVDAIC*QVVR	196.02
tsf	Elongation factor Ts	32.511	TGAGMMDC*K	78.324
xpt	Xanthine phosphoribosyltransferase	20.884	LEEAGLTVSSLC*K	79.332
mqo2	Probable malate : quinone oxidoreductase 2	55.998	EGC*MNHLR	122.94
gmk	Guanylate kinase	24.037	IQC*IVEAEHLK	100.11
HMPREF0776_1272	Mu transposase domain protein	32.351	NDTNWPVC*GIPEK	69.314
MW1126	Ribosome biogenesis GTPase A	33.403	IGNYC*FDIFK	65.809
fabG	β-Ketoacyl-ACP reductase	24.616	GVFNC*IQK	121.73
SAKOR_02572	Transcriptional regulator, TetR family protein	22.356	ALLQC*IEAGNNK	89.484
SAKOR_02572	Transcriptional regulator, TetR family protein	22.356	SDLC*YYVIQR	129.82
nth	Endonuclease III	25.668	LC*SVIPR	78.264
SA0511	Uncharacterized epimerase/dehydratase	36.052	QGIANSWPDSIDTSC*SR	155.06
SA0511	Uncharacterized epimerase/dehydratase	36.052	VAGELLC*QYYFK	119
tagF_2	Putative teichoic acid biosynthesis protein	45.954	IC*QTLFK	66.056
mnmG	tRNA uridine 5-carboxymethylaminomethyl modification enzyme MnmG	69.507	YC*PSIEDK	131.09
rpoA	DNA-directed RNA polymerase subunit alpha	35.011	SYNC*LKR	61.593
tagB	Teichoic acid biosynthesis protein B	18.212	C*LPGYTLINK	116.74
SAOUHSC_01812	Uncharacterized protein	35.096	C*IEDNDTIIIHR	141.34
SAV1710	Putative universal stress protein SAV1710	18.38	HAPC*DVLVVR	131.83
srtA	LPXTG-specific sortase A	23.541	QLTLITC*DDYNEK	152.32
ST398NM01_1322	Uncharacterized protein	28.118	SELEELC*K	118.23
recG	ATP-dependent DNA helicase RecG	78.343	C*IFFNQPYLK	125.72
SAKOR_00906	Oligopeptide transport ATP-binding protein oppF	16.074	GETLGLVGESGC*GK	102.03
murD	MURD	29.82	NQTEEDYLIC*NYHQR	92.151
SA0315	Uncharacterized protein	20.923	C*DAPMEVNK	73.435
HMPREF0776_0655	3-Demethylubiquinone-9 3-methyltransferase domain protein	16.768	VLC*SDSFGR	132.03
SAOUHSC_02324	Uncharacterized protein	24.634	QFFTEWNC*HD	141.58
rnc	Ribonuclease III	27.922	ATIVC*EPSLVIFANK	95.273
topA	DNA topoisomerase I	79.07	C*NDGDVVER	92.151
topA	DNA topoisomerase I	79.07	C*NQYLVENK	126.12
rpmJ	50S ribosomal protein L36	4.3	VMVIC*ENPK	183.99
SAZ172_2022	Uncharacterized protein	24.952	TVIIDC*VTK	109.44
N/A	Uncharacterized protein	13.573	GIDLNMGC*PVANVAK	167.93
polC_2	DNA polymerase III PolC-type	4.1918	TSIC*SVGMVK	67.952
ackA	Acetate kinase	44.056	IISC*HIGNGASIAAIDGGK	50.404
SAOUHSC_02264	Accessory gene regulator protein C	13.754	C*ADDIPR	101.46
rnpA	Ribonuclease P protein component	11.093	QFVVYTC*NNK	166.48
rpmG	50S ribosomal protein L33	5.87	VNVTLAC*TEC*GDR	134.08
rpmG	50S ribosomal protein L33	5.87	VNVTLAC*TEC*GDR	100.01
N/A	mRNA interferase PemK	20.91	FLC*DSLK	100.25
graR	Response regulator protein GraR	26.066	YDGFYWC*R	123.79
SAKOR_02101	Uncharacterized protein	42.899	VVVLGC*PAEEGGENGSAK	95.755
SAKOR_02241	Molybdopterin biosynthesis MoeB protein	37.894	YATLC*GR	87.806
cshB	DEAD-box ATP-dependent RNA helicase CshB	51.064	C*NAQPQLIIGTPTR	86.016
SACOL0939	NifU domain protein	7.9651	DGGDC*SLIDVEDGIVK	163.45
SAKOR_00091	Ornithine cyclodeaminase family protein	37.776	VVVDDWSQC*NR	91.592
SA2277	Uncharacterized protein	50.847	TILC*ALDVR	107.34
glnA	Glutamine synthetase	50.854	GFTAVC*NPLVNSYK	229.96
glnA	Glutamine synthetase	50.854	LIC*DVYK	111.61
glnA	Glutamine synthetase	50.854	LVPGYEAPC*YIAWSGK	134.32
glnA	Glutamine synthetase	50.854	YADAVTAC*DNIQTFK	154.34
whiA	Putative sporulation transcription regulator WhiA	35.868	LVNC*ETANLNK	154.72
whiA	Putative sporulation transcription regulator WhiA	35.868	NNIYIC*R	157.91
SAKOR_00683	Transcriptional regulator, MarR family protein	17.089	EQLC*FSLYNAQR	207.66
menB	1,4-Dihydroxy-2-naphthoyl-CoA synthase	30.411	EIWYLC*R	154.12
menB	1,4-Dihydroxy-2-naphthoyl-CoA synthase	30.411	VEDETVQWC*K	185.57
perR	Peroxide-responsive repressor PerR	17.183	MEIYGVC*K	120.45
SAOUHSC_00462	Uncharacterized protein	29.281	GLSYEEVC*EQTTK	170
tpx	Probable thiol peroxidase	18.005	LISVVPSIDTGVC*DQQTR	81.656
SAOUHSC_00960	Uncharacterized protein	57.927	VTMTDYC*YR	87.216
ybhF_3	ABC transporter ATP-binding protein	32.952	LEDIELIC*DR	121.42
BN1321_100031	MutT/NUDIX family protein	15.067	C*VCLVEETADK	193.28
SAKOR_00705	Uncharacterized protein	14.205	YDEVTIYC*K	153.74
SAOUHSC_01677	Uncharacterized protein	25.002	TAQTVTDLRPAGIIFC*ENER	111.77
SAKOR_00374	Phosphoglycerate mutase family protein	22.777	SADDLC*DYFK	121.56
sufB	Fe–S cluster assembly protein SufB	52.531	YPNC*VLLGEGAK	121.45
ccpA	Catabolite control protein A	12.161	NGLQLGDTLNC*SGAESYK	140.07
SAV0485	Signal peptidase II-like protein	23.935	DAENALILC*K	85.672
SAV0485	Signal peptidase II-like protein	23.935	LVNC*EYTLDK	97.223
sucD	Succinate-CoA ligase [ADP-forming] subunit alpha	31.542	LVGPNC*PGVITADEC*K	249.56
sucD	Succinate-CoA ligase [ADP-forming] subunit alpha	31.542	LVGPNC*PGVITADEC*K	179.85
sucD	Succinate-CoA ligase [ADP-forming] subunit alpha	31.542	TLNSC*GVK	91.469
SAKOR_01205	Transcriptional regulator, GntR family protein	26.976	EQSNHNIC*YADTEIEAVNYEPR	97.621
SAKOR_01205	Transcriptional regulator, GntR family protein	26.976	TADGEPVVYC*LDK	129.88
SAOUHSC_02755	Uncharacterized protein	39.192	YGC*ALAIEVLK	79.283
ugtP	Processive diacylglycerol β-glucosyltransferase	44.547	SANAQVVMIC*GK	213.99
ugtP	Processive diacylglycerol β-glucosyltransferase	44.547	YATQTIC*R	122.18
MW0660	MW0660 protein	28.342	TGC*SASTIR	104.07
MW0924	Uncharacterized protein	18.511	SC*VDATYR	89.171
MW0924	Uncharacterized protein	18.511	VAGC*IISYSGENELK	145.17
MW0924	Uncharacterized protein	18.511	WSLNC*DINNEAALK	126.04
SAOUHSC_01973	Uncharacterized protein	35.778	EAEILC*YIDNIDAR	77.505
SAOUHSC_01973	Uncharacterized protein	35.778	SIC*DIYPLLNK	112.44
upp	Uracil phosphoribosyltransferase	23.05	FMC*LIAAPEGVEK	131.96
SAOUHSC_02727	Uncharacterized protein	22.28	C*IYVQPHSYTIENQQQNK	119.37
MW0535	MW0535 protein	29.857	AGEVYEASNAQYFVVDPVMVC*K	55.621
HMPREF0776_0347	CHAP domain protein	12.753	NLYTSGQC*TYYVFDR	78.615
SAOUHSC_02574	Uncharacterized protein	40.742	SCLNDC*YDK	139.74
SAV2378	Uncharacterized protein	13	C*ANEEER	63.624
tagH_1	Teichoic acids export ATP-binding protein TagH	29.762	MLC*MGFK	101.28
CH52_06005	2-Dehydropantoate 2-reductase	32.358	QLLLDGC*R	82.279
glyS	Glycine–tRNA ligase	53.62	IIDDEGIVC*PVSK	195.45
glyS	Glycine–tRNA ligase	53.62	YIPYC*IEPSLGADR	107.98
tpiA	Triosephosphate isomerase (TIM) (TPI)	27.261	HGMTPIIC*VGETDEER	135.95
tpiA	Triosephosphate isomerase (TIM) (TPI)	27.261	SSTSEDANEMC*AFVR	142.93
SAOUHSC_01716	Uncharacterized protein	47.655	C*TLSNHMTAR	60.901
SAOUHSC_01716	Uncharacterized protein	47.655	TGATGIIVADPLIIETC*K	92.8
vraS_1	Histidine kinase (nitrate/nitrite sensor protein) (EC 2.7.3.-) (two-component sensor protein)	41.88	ALQEC*INNVK	101.61
vicR	DNA-binding response regulator (PhoP family transcriptional regulator)	27.192	DGMEVC*R	117.81
SAOUHSC_02218	Conserved hypothetical phage protein	11.1	EISNGHC*NYWK	144.51
SAOUHSC_01696	Uncharacterized protein	22.463	TIDC*LNYYNYSDER	154.72
hsdS_2	Restriction endonuclease subunit S	23.781	IPC*LTEQDK	102.87
sufA	Chaperone involved in Fe–S cluster assembly	12.485	VAGNPENC*	106.16
SAKOR_00641	Ferrichrome transport ATP-binding protein fhuC	29.496	TGKPLLVTYDLC*R	90.755
SAKOR_00641	Ferrichrome transport ATP-binding protein fhuC	29.496	VTSIIGPNGC*GK	164.66
HMPREF0769_12132	ROK family protein	35.077	IILAADVGGTTC*K	103.13
nadK	NAD kinase	30.769	GDGLC*VSTPSGSTAYNK	102.24
mraZ	Transcriptional regulator MraZ	17.237	EC*TVIGVSNR	109.16
yutD	Uncharacterized protein conserved in bacteria	15.401	EC*FNEEQFIAR15.401	149.35
SAZ172_0295	Uncharacterized protein	15.751	C*FEEEDFER	164.48
SAZ172_0295	Uncharacterized protein	15.751	YIDC*LEVGPTLSTK	170
gatA	Glutamyl-tRNA(Gln) amidotransferase subunit A	52.82	DNIITNGLETTC*ASK	128.66
MW0675	MW0675 protein	22.322	YHSLIADGATFPNC*LK	80.905
rpsR	30S ribosomal protein S18	9.3098	VC*YFTANGITHIDYK	100.69
fusA	Elongation factor G	64.009	DTGTGDTLC*GEK	188
fusA	Elongation factor G	64.009	KC*DPVILEPMMK	134.61
fusA	Elongation factor G	64.009	KEFNVEC*NVGAPMVSYR	171.39
fusA	Elongation factor G	64.009	QATTNVEFYPVLC*GTAFK	81.594
rocD2	Ornithine aminotransferase 2	43.417	EEGLLC*K	133.86
hutU	Urocanate hydratase	60.632	GLSIEC*K	80.231
narH	NarH protein	59.446	RDEDGIVLVDQDAC*R	95.988
SAOUHSC_00882	Uncharacterized protein	15.517	LTIIDPHETFC*QR	93.839
SAOUHSC_02811	Uncharacterized protein	27.165	EC*ATEITEVEDK	115.68
NWMN_2186	Acyl-CoA dehydrogenase-related protein	34.413	METLLLC*AR	163.9
fabF	3-Oxoacyl-[acyl-carrier protein] synthase 2	42.433	ALSTNDDIETAC*R	94.61
fabF	3-Oxoacyl-[acyl-carrier protein] synthase 2	42.433	GPNGATVTAC*ATGTNSIGEAFK	70.501
N/A	Putative uncharacterized protein	24.022	VDMIAC*EDTR	92.295
ppaC	Probable manganese-dependent inorganic pyrophosphatase	34.068	AEPVGC*TATILYK	136.26
ppaC	Probable manganese-dependent inorganic pyrophosphatase	34.068	IANFETAGPLC*YR	229.02
ppaC	Probable manganese-dependent inorganic pyrophosphatase	34.068	SPTC*TQQDVK	163.33
AYM28_13750	Nicotianamine synthase	31.096	SLQYITAQC*VK	120.21
HMPREF0769_12162	Transketolase, pyridine binding domain protein	36.033	SNNDWQC*PLTIR	110.57
nos	Nitric oxide synthase oxygenase	41.71	EC*HYETQIINK	55.899
nos	Nitric oxide synthase oxygenase	41.71	YAGYDNC*GDPAEKEVTR	147.84
SAKOR_00998	Hydroxymethylpyrimidine transport ATP-binding protein	53.302	VLLLGPSGC*GK	54.898
SAOUHSC_01872	Uncharacterized protein	46.176	C*SQFVYK	68.657
SAV2122	Putative aldehyde dehydrogenase SAV2122	51.968	VVNNTGQVC*TAGTR	225.69
SAOUHSC_02064	Phi ETA orf 25-like protein	15.401	DVNLTWIC*K	79.089
HUNSC491_pPR9_p11	ATP-binding protein p271 (ATP-binding protein, putative)	7.4555	YQYIGIC*YGQPGVGK	91.065
AYM28_02415	Acetyltransferase (GNAT) family protein	19.908	AQEYSTVVVDHC*FDYFEK	87.963
accD	Acetyl-coenzyme A carboxylase carboxyl transferase subunit β	31.52	IIDYC*TENR	143.42
ldh2	l-lactate dehydrogenase 2 (l-LDH 2)	34.42	AGEYEDC*KDADLVVITAGAPQKPGETR	129.82
gltX	Glutamate–tRNA ligase	18.695	C*YMTEEELEAER	204.18
srpF	Alpha-helical coiled-coil protein	19.257	TYVC*EDMSK	193.22
mnmA	tRNA-specific 2-thiouridylase MnmA	42.15	DSTGIC*FIGEK	84.173
mnmA	tRNA-specific 2-thiouridylase MnmA	42.15	TPNPDVMC*NK	145.52
V070_01284	Uncharacterized protein	48.871	LPYTLC*YISR	145.61
tmk	Uncharacterized protein	51.081	AQLIEC*LEK	79.886
trxA_1	Thiol reductase thioredoxin	11.454	IDLNFYPQFC*K	83.204
pdxT	Pyridoxal 5′-phosphate synthase subunit PdxT	20.63	VGQGVDILC*K	126.71
mcsB	Protein-arginine kinase	38.61	SLGILQNC*R	63.694
ydaG	General stress protein 26	15.886	EDPELC*VLR	152.7
HMPREF0769_12370	SWIM zinc finger domain protein	15.906	GFNYYQSEC*VINLK	157.73
HMPREF0769_10247	Oxidoreductase, FAD-binding protein	42.831	AFLANKPEIYIC*GGTK	107.15
tarI1	Ribitol-5-phosphate cytidylyltransferase 1	26.656	SILSDAC*K	122.69
SACOL2177	Zinc-type alcohol dehydrogenase-like protein	32.773	QETTEWC*EK	218.02
SAOUHSC_02146	Uncharacterized protein	40.354	ESGC*TVFQGK	93.345
SAOUHSC_02146	Uncharacterized protein	40.354	LILENC*R	125.68
SACOL2396	Uroporphyrinogen III methylase SirB, putative	36.29	INDC*IVEAAR	113.37
glpK	Glycerol kinase	55.625	ATLESLC*YQTR	158.91
glpK	Glycerol kinase	55.625	QTQSIC*SELKQQGYEQTFR	125.84
HMPREF3211_00337	Methyltransferase domain protein	21.763	ALDIGC*GSGLLVEK	55.031
tarJ	Ribulose-5-phosphate reductase 1	38.451	IPEGLTFDHAFEC*VGGR	60.968
MW2550	MW2550 protein	29.096	LLIMC*GK	113.41
gpmI	2,3-Bisphosphoglycerate-independent phosphoglycerate mutase	56.423	AIEAVDEC*LGEVVDK	144.75
SAR1875	Putative membrane protein insertion efficiency factor	8.9865	FYPTC*SEYTR	109.95
serS	Serine–tRNA ligase	48.639	EISSC*.SNC*.TDFQAR	122.29
serS	Serine–tRNA ligase	48.639	FTGQSAC*FR	123.26
serS	Serine–tRNA ligase	48.639	MTGILC*R	123.21
serS	Serine–tRNA ligase	48.639	VILC*TGDIGFSASK	124.45
MW2545	MW2545 protein	25.27	GC*TLILDEAK	83.204
femB	Aminoacyltransferase FemB	49.675	YLQQHQC*LYVK	83.53
mfd	Transcription-repair-coupling factor	134.3	LLC*GDVGYGK	84.605
N/A	Kanamycin nucleotidyl transferase protein	27	IC*YTTSASVLTEAVK	119.62
sdhA	SdhA protein	65.502	EIFDVC*INQK	265.75
sdhA	SdhA protein	65.502	GLFAAGEC*DFSQHGGNR	110.77
HMPREF0769_12639	PHP domain protein	8.9913	ASLQVAC*ENGK	121.95
SAZ172_1861	Ribosomal large subunit pseudouridine synthase D-like protein	31.387	C*VSPTGQR	88.056
NWMN_0748	Uncharacterized protein	28.19	GIVTMC*APMGGK	138.55
SAZ172_0851	Pathogenicity island protein	15.839	IIC*DFSTEREEK	134.38
SAKOR_01965	RecT protein	16.895	NQC*YFIPYGNK	86.772
gtf1	Glycosyltransferase Gtf1	58.273	SSFVTC*YLQNEQK	187.56
fbp	Fructose-1,6-bisphosphatase class 3	76.213	VC*LANLLR	91.087
glmU	Bifunctional protein GlmU [includes: UDP-*N*-acetylglucosamine pyrophosphorylase]	48.532	EGTTIVVC*GDTPLITK	109.28
glmU	Bifunctional protein GlmU [includes: UDP-*N*-acetylglucosamine pyrophosphorylase]	48.532	TNIGC*GTITVNYDGENK	155.26
SAOUHSC_02464	Uncharacterized protein	32.803	NIEAC*TSLK	62.162
trxA_2	Thiol reductase thioredoxin	12.141	FEAGWCPDC*R	119.87
aroC	Chorismate synthase	43.059	VAVGALC*K	123.35
MW0527	MW0527 protein	23.895	AC*GLTEPSSK	168.85
MW0527	MW0527 protein	23.895	C*GEVATQSAFK	97.273
lolD_1	ABC transporter ATP-binding protein	24.698	AC*IIVTHDER	73.435
pckA	Phosphoenolpyruvate carboxykinase	59.377	NGVFNIEGGC*YAK	207.21
glmS	Glutamine–fructose-6-phosphate aminotransferase	65.835	C*GIVGYIGYDNAK	159.41
ST398NM01_0974	Uncharacterized protein	81.447	GELHC*IGATTLNEYR	166.21
SAV0406	Uncharacterized protein	29.041	GWNTLC*TYLK	147.39
SAOUHSC_02980	Uncharacterized protein	20.729	SC*DIESVESWK	130.19
lysA_1	Diaminopimelate decarboxylase	9.757	AFTC*IQMVK	106.26
HMPREF0776_0362	HTH domain protein	26.531	QC*LSLPQTR	63.966
SAKOR_02509	Transcriptional regulator, MarR family protein	16.544	VYMAC*LTEK	137.01
SAOUHSC_00118	Capsular polysaccharide biosynthesis protein Cap5E, putative	38.591	SEQTLIC*GTR	184.51
SAOUHSC_00118	Capsular polysaccharide biosynthesis protein Cap5E, putative	38.591	VIC*LSTDK	157.66
SAOUHSC_02364	Uncharacterized protein	12.686	MEVC*PYLEETFK	158.89
SAOUHSC_02584	Uncharacterized protein	30.385	AC*HETVLK	114.97
SAOUHSC_02584	Uncharacterized protein	30.385	GEGAFC*NGIK	114.72
SAOUHSC_02584	Uncharacterized protein	30.385	LIC*SWLK	118.33
map	Methionine aminopeptidase	27.358	EIGYIC*AK	128.86
QU38_16080	Acyl-CoA ligase	59.748	LGVAIIPC*SEMLR	111.07
AYM28_10805	6-Phosphogluconolactonase	38.546	AGTGC*YVSISEDKR	105.63
AYM28_10805	6-Phosphogluconolactonase	38.546	EGEQC*GVASLK	153
AYM28_10805	6-Phosphogluconolactonase	38.546	ITLC*DNTR	151.13
SAOUHSC_02891	Uncharacterized protein	21.555	SCELNSEAFC*NK	123.72
SA2075	Sulfur carrier protein FdhD	19.75	LYGFC*IQR	106.68
pth	Peptidyl-tRNA hydrolase	21.703	C*IVGLGNIGK	84.31
dnaK	Chaperone protein DnaK	66.361	IIGIDLGTTNSC*VTVLEGDEPK	89.507
SAOUHSC_01064	Pyruvate carboxylase	18.812	C*AEEGIK18.812	77.73
sarR	HTH-type transcriptional regulator SarR	13.669	C*SEFKPYYLTK	98.421
lepA	Elongation factor 4	28.674	C*YGGDISR	128.35
asnS	Asparagine–tRNA ligase	49.157	SVLENC*KLELK	125.97
pfkA	ATP-dependent 6-phosphofructokinase	34.839	C*PEFKEQEVR	108.97
SAKOR_01872	Uncharacterized protein	12.527	FILSTSDDSDYIC*K	91.589
lipA_2	Lipoyl synthase	34.885	HC*QAGPLVR	83.08
lipA_2	Lipoyl synthase	34.885	NLNTVC*EEAK	222.56
V070_00687	Uncharacterized protein	27.971	LINPDC*K	128.35
SAOUHSC_00532	Uncharacterized protein	42.89	NDAILSDELNHASIIDGC*R	87.411
rnj2	Ribonuclease J 2 (RNase J2)	62.603	LIVSC*YASNFIR	118.71
MW1645	MW1645 protein	44.233	C*FEIEER	74.165
miaB_1	MiaB family protein, possibly involved in tRNA or rRNA modification	50.955	STVAFHTLGC*K	79.614
ung	Uracil-DNA glycosylase (UDG)	24.967	ELADDIGC*VR	138.24
glpD	Aerobic glycerol-3-phosphate dehydrogenase	62.387	KDYGLTFSPC*NTK	250.46
SAV0941	NADH dehydrogenase-like protein	44.104	IATPIVAC*NEK	236.05
SAV0941	NADH dehydrogenase-like protein	44.104	IPELC*SK	154.01
MW2452	MW2452 protein	24.558	LDC*KDEFIK	89.189
SA1530	Uncharacterized peptidase	39.606	QVLFC*PK	132.76
pheT_2	Phenylalanine–tRNA ligase β subunit	12.153	GVASSGMIC*SMK	86.772
SAKOR_02579	Putative cytosolic protein	11.547	YMFDYSAC*K	90.614
AYM28_07495	DNA-binding protein	12.72	HYQQLINQC*K	121.29
gcvPB	Probable glycine dehydrogenase (decarboxylating) subunit 2	22.485	NFGVDNGFYPLGSC*TMK	102.15
NWMN_0123	Uncharacterized protein	151.91	SLLEC*VK	99.013
adh	Alcohol dehydrogenase (ADH)	36.061	LDPAAASSITC*AGVTTYK	210.33
dnaJ	DnaJ	29.458	TEQVC*PK	88.495
guaB	Inosine-5′-monophosphate dehydrogenase	52.85	VGIGPGSIC*TTR	119.46
N/A	UPF0413 protein	25.089	C*QAQSTSNFDNIALAYK	125.39
SAKOR_00478	VEG protein	9.9982	NSIDC*HVGNR	76.868
mutS	MutS protein	48.889	SEYQDC*LLFFR	79.885
mutS	MutS protein	48.889	VAIC*EQMEDPK	125.36
yibN	Putative sulfur transferase	14.803	KDQPVYLC*DANGIASYR	78.191
ileS	Isoleucine–tRNA ligase	104.74	C*KEFALEQIELQK	116.61
SAOUHSC_01907	Uncharacterized protein	31.471	VENDENC*MESVK	159.04
nagB	Glucosamine-6-phosphate deaminase	28.467	QASFYVAC*ELYK	106.82
SAKOR_02240	Molybdenum cofactor biosynthesis protein B	18.5	DFDTDKGGQC*VR	93.716
nirB	Nitrite reductase [NAD(P)H], large subunit	46.979	SC*VESGVK	65.627
tetM	Tetracycline resistance protein TetM	70.346	GPSELC*GNVFK	69.261
thyA	Translation initiation factor IF-3	36.825	LSC*QLYQR	55.721
SAOUHSC_01781	Uncharacterized protein	36.431	FANC*TQELTIEK	110.6
miaB	Uncharacterized protein	58.916	AWVNIMYGC*DK	135.91
miaB	RNA methyltransferase TrmA family protein	58.916	YEGQTVTVLC*EGSSK	188.32
pgcA	Fructose-1,6-bisphosphate aldolase	62.376	C*PNFDDVAQK	151.3
SAKOR_02003	Ribosomal protein-serine acetyltransferase	27.906	C*HNSFVVNR	98.407
SAKOR_02003	Methylenetetrahydrofolate–tRNA-(uracil-5-)-methyltransferase TrmFO	27.906	IFIC*EDDPK	142.19
SAKOR_02003	Methylenetetrahydrofolate–tRNA-(uracil-5-)-methyltransferase TrmFO	27.906	IIDC*LETAHTR	170.95
SAV1153	Methylenetetrahydrofolate–tRNA-(uracil-5-)-methyltransferase TrmFO	19.255	GNC*DFYPEFENEAVAK	76.341
ftsH	Peptide methionine sulfoxide reductase MsrB	77.812	IC*GLLGGR	124.08
HMPREF0769_10485	Putative peptide methionine	18.28	LDSPYDGYAEC*VK	115.26
sepF	Cell division protein SepF	20.686	MC*LFEPR	120.15
SAOUHSC_02898	Uncharacterized protein	24.931	VNSLAYC*SSK	128.85
sucC	Succinate-CoA ligase [ADP-forming] subunit β	42.056	C*DVIAEGIVEAVK	189.04
sucC	Succinate-CoA ligase [ADP-forming] subunit β	42.056	RLYIEEGC*AIQK	231.07
SA2102	Putative formate dehydrogenase	111.29	FAEEC*AK	160.82
SA2102	Putative formate dehydrogenase	111.29	GHNNVQGC*SDMGSMPDK	88.108
SA2102	Putative formate dehydrogenase	111.29	QVIGTNNVDNC*SR	175.19
SA2102	Putative formate dehydrogenase	111.29	YC*QAPATK	117.25
SAKOR_00737	Ferric anguibactin transport ATP-binding protein	28.62	STLLSAIC*R	103.43
SAOUHSC_01323	Uncharacterized protein	29.821	QDFDEIVDYC*R	127.05
SAOUHSC_02248	Uncharacterized protein	17.196	IIGLSGMC*K	68.132
taqD	Glycerol 3-phosphate cytidyltransferase	15.789	C*EVIYLK	51.528
AYM28_05950	Uncharacterized protein	15.185	FQMINDC*AEK	64.52
queA	*S*-adenosylmethionine:tRNA ribosyltransferase-isomerase	38.97	IIAEC*IK	138.08
SAOUHSC_00086	3-Ketoacyl-acyl-carrier protein reductase, putative	27.215	IINATSQAGVEGNPGLSLYC*STK	70.572
glcT	Protein GlcT	32.822	NHYPIC*YNTAYK	118.52
AYM28_01135	AraC family transcriptional regulator	29.599	VVIC*DDER	129.82
AYM28_01135	AraC family transcriptional regulator	29.599	YLQMSPSDYC*K	115.09
AYM28_13045	Putative 3-methyladenine DNA glycosylase	22.771	AIDGATLNDC*R	116.58
tyrC	Arogenate dehydrogenase	40.395	C*LNYSEAIK	68.676
SAOUHSC_02899	Uncharacterized protein	38.194	AIELC*QK	143.37
SAR2150	Protein SprT-like	17.186	ANYEYYC*TK	166.62
SAR2150	Protein SprT-like	17.186	FC*NSIESYQQR	97.797
SA0314	Uncharacterized protein	20.027	LDC*AEIIR	73.841
SA1974	Probable uridylyltransferase	44.865	LVNVDC*K	82.417
hemH	Ferrochelatase	35.056	VVC*DDIGANYYRPK	112.24
hpt	Hypoxanthine-guanine phosphoribosyltransferase	20.154	EVLLTEEDIQNIC*K	103.39
SAOUHSC_00548	Uncharacterized protein	58.418	GFLSC*SR	94.309
SAOUHSC_00531	Uncharacterized protein	43.657	VRPGAFFLTGC*GNESK	74.296
tnp	Putative transposase	8.5839	GIEC*IYALYK	90.614
cap5G	Capsular polysaccharide biosynthesis protein Cap5G	42.851	C*FDQNVPEEINR	186.96
SAOUHSC_00973	Uncharacterized protein	27.727	VC*YQVFYDEK	138.02
ykaA	Phosphate transport regulator	22.598	EFETNC*DGILR	133.48
hprK	HPr kinase/phosphorylase	34.481	LC*RPETPAIIVTR	98.508
murA1	UDP-*N*-acetylglucosamine 1-carboxyvinyltransferase 1	44.995	LGHAIVALPGGC*AIGSR	67.846
rocA	1-Pyrroline-5-carboxylate dehydrogenase	56.867	GC*TSAVVGYHPFGGFK	103.67
HMPREF0769_10271	Oxidoreductase, NAD-binding domain protein	39.204	AAC*AAEAYGTDNAK	78.917
ahpC	Alkyl hydroperoxide reductase	20.976	KNPGEVC*PAKWEEGAK	127.78
mvaS	HMG-CoA synthase	43.217	EAC*YAATPAIQLAK	226.7
SAKOR_00677	Cytokinin riboside 5′-monophosphate phosphoribohydrolase	20.889	ALAPLC*DTK	137.4
SAKOR_00677	Cytokinin riboside 5′-monophosphate phosphoribohydrolase	20.889	IAVYC*GASK	122.33
pcrA	ATP-dependent DNA helicase PcrA	84.073	IC*YVAITR	109.83
N/A	Putative uncharacterized protein	15.429	EQGSDIDAAC*GQLR	137.52
rimP	Ribosome maturation factor RimP	17.627	EGGVDLNDC*TLASEK	197.6
asp1	Accessory Sec system protein Asp1	53.78	EC*ITSVNEEYQAK	188
SAUSA300_2158	Uncharacterized protein	14.457	DDNILC*EEFSYK	79.393
SAKOR_00594	Trp repressor-binding protein	20.243	VILVGGDC*PK	186.76
SAOUHSC_02447	Uncharacterized protein	36.266	VPVC*GAISSYNHPEADIGPR	126.62
SAOUHSC_00497	Uncharacterized protein	53.954	NGLTLQEC*LDR	114.64
SAOUHSC_00497	Uncharacterized protein	53.954	SIFPSC*R	68.557
pyk	Pyruvate kinase	63.102	C*DILNSGELK	126.71
pyk	Pyruvate kinase	63.102	IVC*TIGPASESEEMIEK	96.561
pyk	Pyruvate kinase	63.102	QC*SIVWGVQPVVK	172.11
rpoB	DNA-directed RNA polymerase subunit β	133.22	FMDDEVVC*R	92.247
yigZ	ABC transporter	23.868	EAVPC*IVTLNYDQTGK	120.21
yigZ	ABC transporter	23.868	LDVHNAC*VVVTR	98.182
N/A	Uncharacterized protein	32.909	NESLC*ELKK	140.63
SAOUHSC_01365	Uncharacterized protein	37.855	SHLVNLC*K	99.788
SA2162	Ferredoxin–NADP reductase	38.164	C*NTLLSETSSK	186.08
SA2162	Ferredoxin–NADP reductase	38.164	LDMHDDC*R	64.224
infC	Translation initiation factor IF-3	20.244	YADEC*KDIATVEQKPK	88.311
SAOUHSC_02827	Uncharacterized protein	10.548	IIASC*SFAK	135.1
SAOUHSC_02579	Uncharacterized protein	41.89	VLYQGYTC*FR	109.99
NWMN_1835	RNA methyltransferase TrmA family protein	51.682	IVYISC*NPATQQR	216.34
fba	Fructose-bisphosphate aldolase	30.836	EC*QELVEK	97.472
SAKOR_00338	Ribosomal protein-serine acetyltransferase	20.294	YC*FEELDLNR	77.644
trmFO	Methylenetetrahydrofolate–tRNA-(uracil-5-)-methyltransferase	48.371	FAELVC*SNSLR	144.4
trmFO	Methylenetetrahydrofolate–tRNA-(uracil-5-)-methyltransferase	48.371	YDKGEAAYLNC*PMTEDEFNR	90.259
trmFO	Methylenetetrahydrofolate–tRNA-(uracil-5-)-methyltransferase	48.371	YFEGC*MPFEVMAER	85.937
msrB	Peptide methionine sulfoxide reductase MsrB	16.277	FHSEC*GWPSFSK	125.33
msrB	Peptide methionine sulfoxide reductase MsrB	16.277	YC*INSAAIQFIPYEK	144.3
ytqA	Fe–S oxidoreductase	36.053	VALDGGFDC*PNR	116.87
yjlD	NADH dehydrogenase	39.399	IYNC*DEPK	160.72
SAZ172_2586	Mutator mutT protein	11.465	C*DLIVGDK	63.073
AYM28_03635	Protein of uncharacterized function	14.312	IMYC*FNK	132.08
SAKOR_01397	ATP-dependent helicase, DinG family protein	104.19	C*LVLFTSYK	112.13
SAOUHSC_02393	Uncharacterized protein	25.34	C*LANNDVQIMNSIK	78.674
panD	Aspartate 1-decarboxylase	14.05	IC*LNGAASR	112.64
purA	Adenylosuccinate synthetase	47.551	IC*TAYELDGK	104.59
fhs	Formate–tetrahydrofolate ligase	59.871	QFKENGWDNYPVC*MAK	200
SAZ172_2084	5-Amino-6-(5-phosphoribosylamino)uracil reductase	15.666	AFQILHEQYGC*K	51.727
gatB_2	Aspartyl/glutamyl-tRNA(Asn/Gln) amidotransferase subunit B	53.656	C*DANISLRPYGQEK	76.574
SAZ172_2659	d-specific d-2-hydroxyacid dehydrogenase-like protein	37.264	DAVFVNC*AR	73.435
folP	Dihydropteroate synthase	29.532	SEVAEAC*LK	115.33
AYM28_03210	Deoxyguanosinetriphosphate triphosphohydrolase	50.595	GGEVLLNNC*LK	164.22

Furthermore, the prevalence of hydrophobic and positively charged amino acids flanking modified cysteines was observed when linear amino acid sequences and available 3D structures of CoAlated proteins were examined using computational methods (manuscript in preparation).

To sense and overcome the oxidative stress, *S. aureus* employs oxidation-sensing transcriptional regulators, such as MgrA, SarZ and SarA, and the quorum-sensing Agr system which controls global gene expression via the redox-active Cys residues [44,45]. It was interesting to find redox-sensing transcriptional regulators detected among CoAlated proteins. In total, 16 CoA-modified peptides from 12 transcriptional regulators, including SarR, CtsR, AgrA, PerR and SarS, were found in diamide-treated *S. aureus*. Induction of the CtsR and PerR regulons in NaOCl-treated *B. subtilis* was found to be indicative of the disulfide stress response [46]. PerR exists in Gram-positive bacteria as a functional homolog of OxyR and functions as a sensor of oxidative stress. PerR possesses four cysteine residues, which are involved in Zn coordination, dimerization and DNA binding. One of these cysteines, Cys142, was found to be CoAlated in diamide-treated *S. aureus*. The effect of PerR CoAlation on its DNA-binding and transcriptional activities in response to oxidative and metabolic stress remains to be investigated.

The quorum-sensing transcriptional regulator AgrA was CoAlated on Cys6 and Cys199 in diamide-treated cells. The oxidation-sensing role of Cys199 in AgrA was revealed in mutational, biochemical and mass spectrometric analyses [47]. The formation of the intracellular disulfide bond between Cys199 and Cys228 was shown to cause the dissociation of AgrA from DNA. It would be interesting to examine the pattern of AgrA CoAlation in *S. aureus* exposed to different stresses, including nitrogen and carbohydrate deprivation, exposure to heat, UV and oxidizing chemicals. Furthermore, *in vitro* CoAlation of recombinant AgrA at Cys199 has the potential to modify its DNA-binding and transcriptional activities.

Other targets of protein CoAlation are antioxidant proteins, including thioredoxin (Trx), alkyl hydroperoxide reductase C (AhpC), thiol peroxidase (Tpx), malate : quinone oxidoreductases 1 and 2 (Mqo1/2), FAD-binding protein oxidoreductase (HMPREF0769_10247) and Fe–S oxidoreductase (YtqA). In Tpx, diamide-induced CoAlation occurs on the active site Cys60 and the relevance of this modification is yet to be fully understood. Interestingly, Tpx peroxidase was shown to be S-mycothiolated at Cys60 in *Corynebacterium glutamicum* and its activity inhibited by *in vitro* S-mycothiolation [42].

In addition, many ribosomal proteins were found to be CoAlated, including L12, S12, L14, S18, L32, L33 and L36. Recently, ribosomal proteins L33 (RpmG3) and L36 (RpmJ) were found to be S-bacillithiolated within the CXXC motifs at conserved Zn-binding sites in *S. aureus* treated with NaOCl [25]. Oxidation of cysteines in ribosomal proteins, especially in the CXXC motif, was reported in various studies, and it has been suggested that disulfide stress could lead to a stalling of the ribosomes and thus, to the release of the alarmone ppGpp [48,49]. Furthermore, glutathionylation of ribosomal protein S12 was found in oxidatively stressed human T lymphocytes, but the importance of this modification has not been investigated [50]. The identification of many ribosomal proteins as targets for CoAlation may suggest the inhibitory effect of this modification on protein biosynthesis under oxidative stress.

The largest functional group of CoAlated proteins includes metabolic enzymes that function in diverse anabolic and catabolic pathways for carbohydrates, amino acids, nucleotides, fatty acids, coenzymes and antioxidants (Figure 4B and Table 1). Among CoAlated proteins, we found key players of the citric acid cycle, glycolysis, gluconeogenesis, glycerol catabolism and the glyoxylate shunt. These included glyceraldehyde-3-phosphate dehydrogenase 2 (GapA2), pyruvate kinase (Pyk), ATP-dependent 6-phosphofructokinase (PfkA), acetate kinase (AckA), alcohol dehydrogenase (Adh), aldehyde dehydrogenase 1 (AldA1), triose phosphate isomerase (TpiA), manganese-dependent inorganic pyrophosphatase (PpaC), fructose-1,6-bisphosphate aldolase (Fbp), glycerol kinase (GlpK), inosine-5′-monophosphate dehydrogenase (GuaB), malate dehydrogenase (Mdh) and others. In the list of CoAlated metabolic enzymes, we found several enzymes, which are known to be modified by other LMW thiols, including glutathione, bacillithiol and mycothiol. For example, GAPDH, GuaB and AldA were shown to be S-bacillithiolated in *S. aureus* under NaOCl stress [25]. Mycothiolation of GuaB and Fbp in *C. glutamicum* treated with NaOCl was previously reported [34]. In Gram-negative bacteria, GAPDH, TpiA and PpaC were shown to be glutathionylated in oxidative stress response [51,52]. Extensive CoAlation of metabolic enzymes in response to oxidative stress may reflect the potential involvement of CoA in regulatory and/or feedback mechanisms which control metabolic pathways by balancing the redox state.

### *In vivo* CoAlation of SaGAPDH in response to various oxidizing agents

**Figure 5.**
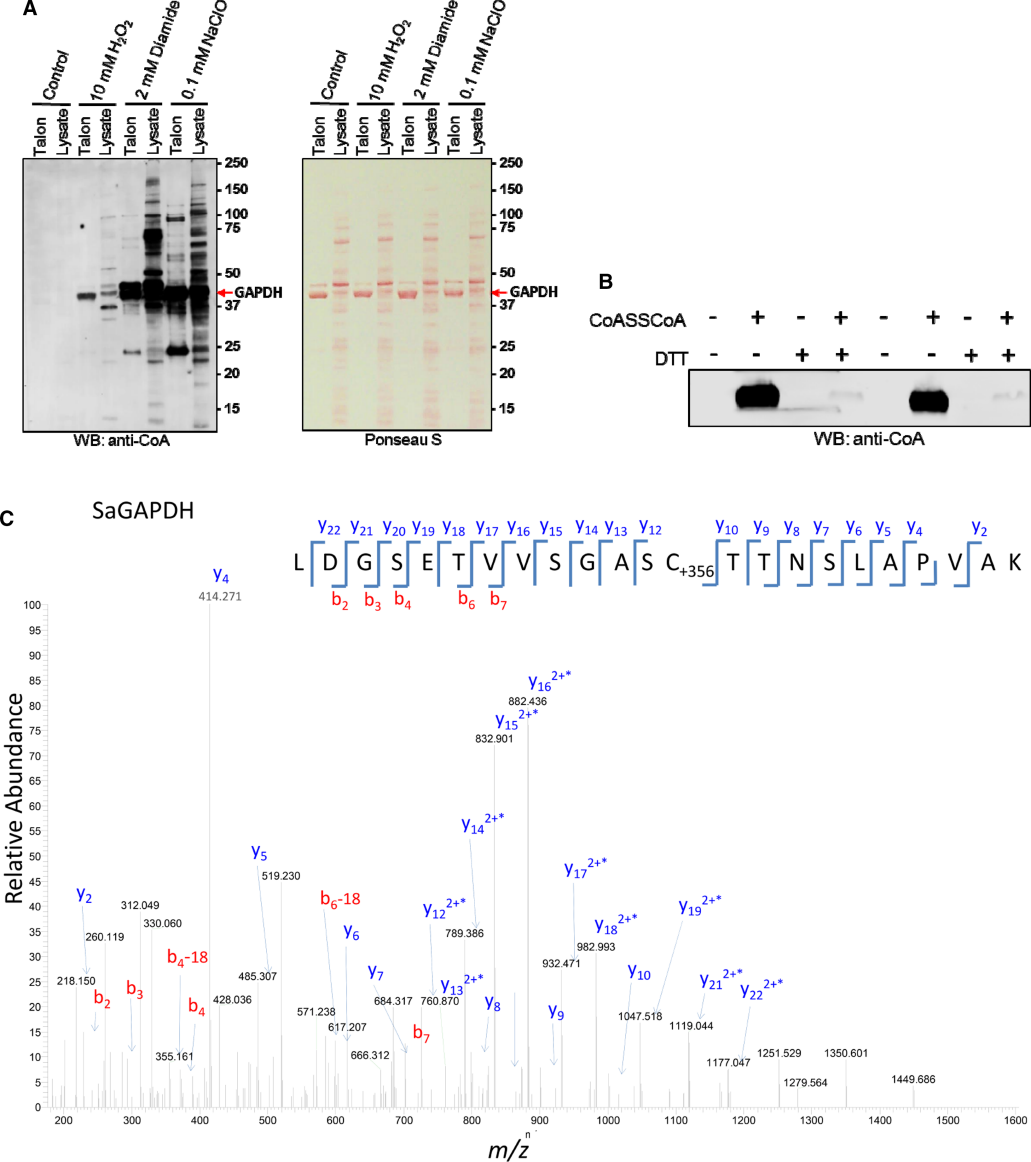
*In vitro* and *in vivo* CoAlation of SaGAPDH. (**A**) CoAlation of SaGAPDH overexpressed in *E. coli* is strongly induced by oxidizing agents. The expression of His-tagged SaGAPDH in *E. coli* transformed with the pQE3/SaGAPDH plasmid was induced with 0.1 mM IPTG for 3 h at 30°C. Bacterial cultures were then treated with 2 mM diamide, 10 mM H_2_O_2_ and 100 µM NaOCl for 30 min. Ni-NTA Sepharose was used to pull-down His-SaGAPDH and protein CoAlation analyzed by immunoblotting with anti-CoA antibody. (**B**) *In vitro* CoAlation of recombinant SaGAPDH. Recombinant preparations of His-SaGAPDH were incubated with 2 mM CoA dimer (CoASSCoA). NEM (25 mM) was added and samples were heated in loading buffer with or without DTT. CoAlation of enzymes was examined by anti-CoA immunoblot. (**C**) LC–MS/MS spectrum of a CoAlated peptide derived from *in vitro* CoAlated SaGAPDH. The spectrum shows a peptide from SaGAPDH (LDGSETVVSGASC*TTNSLAPVAK), containing CoAlated catalytic cysteine 151.

GAPDH homologs in eukaryotic and prokaryotic cells have been found in numerous studies as targets for oxidation and subjects for redox-controlled post-translational modifications, including S-glutathionylation, bacillithiolation and mycothiolation. These modifications were mapped to a strictly conserved catalytic site cysteine and shown to inhibit the activity of GAPDH, and to protect the catalytic cysteine from irreversible overoxidation. Two homologs of GAPDH have been identified in *S. aureus*, termed GAPDH1 and GapA2. In the present study, diamide-induced CoAlation was shown to modify the GapA2 isoform at a non-catalytic Cys202. Protein sequence analysis revealed that trypsin/Lys C digestion of SaGAPDH and GapA2 would produce very long peptides, containing catalytic active cysteines 151 and 153, respectively, making their analysis by LC–MS/MS less feasible. To date, detailed structure–function analysis has been carried out mainly for the SaGAPDH isoform. Therefore, we investigated SaGAPDH CoAlation *in vitro* and *in vivo*, and examined the effect of this modification on its activity. Initially, our efforts were focused on validating *in vivo* CoAlation of SaGAPDH in response to diamide and testing the effect of other oxidizing agents. To do so, *E. coli* transformed with the pQE3/SaGAPDH plasmid were grown at 37°C in LB medium to mid-log phase (OD_600_ = 0.7) and induced with 0.1 mM IPTG for 3 h at 30°C. Bacterial cultures were then treated for 30 min at 37°C with 2 mM diamide, 10 mM H_2_O_2_ and 100µM NaOCl. Ni-NTA Sepharose was used to capture His-SaGAPDH from lysed cells and the pulled-down proteins were analyzed by SDS–PAGE and immunoblotting with anti-CoA antibody. As shown in Figure 5A (right panel), the level of pulled-down His-SaGAPDH was nearly the same in all examined samples. No CoAlation of His-SaGAPDH was detected in control cells, while the treatment of cells with 2 mM diamide induced strong CoAlation of His-SaGAPDH. The strongest CoAlation of His-SaGAPDH was observed in response to NaOCl and a weak immunoreactive signal was detected in H_2_O_2_-treated cells.

Next, we investigated the effect of *in vitro* CoAlation on the activity of SaGAPDH. In the present study, recombinant His-SaGAPDH was purified by Ni-NTA Sepharose chromatography from *E. coli* transformed with the pQE3/His-SaGAPDH plasmid. To produce CoAlated SaGAPDH, an *in vitro* CoAlation assay was performed in the presence of recombinant SaGAPDH and CoASSCoA. Immunoblotting of reaction mixtures with the 1F10 antibody revealed a strong immunoreactive signal corresponding to the SaGAPDH sample incubated with CoA disulfide (Figure 5B). No immunoreactivity was detected in the sample separated under reducing conditions (100 mM DTT). The LC–MS/MS analysis of *in vitro* CoAlated SaGAPDH showed that catalytic Cys151 is CoAlated in the LDGSETVVSGASC*TTNSLAPVAK peptide (Figure 5C).

### *In vitro* CoAlation of SaGAPDH prevents irreversible inhibition of its enzymatic activity by H_2_O_2_

GAPDH homologs in eukaryotic and prokaryotic cells have been shown to be readily inhibited by a variety of ROS and the inhibitory effect is mediated by direct oxidation of the catalytic active cysteine located in a highly conserved CTTNC motif. Studies from several laboratories revealed that SaGAPDH is very sensitive to irreversible oxidation to sulfonic acid when *S. aureus* is treated with 100 mM H_2_O_2_ [25,53].

**Figure 6.**
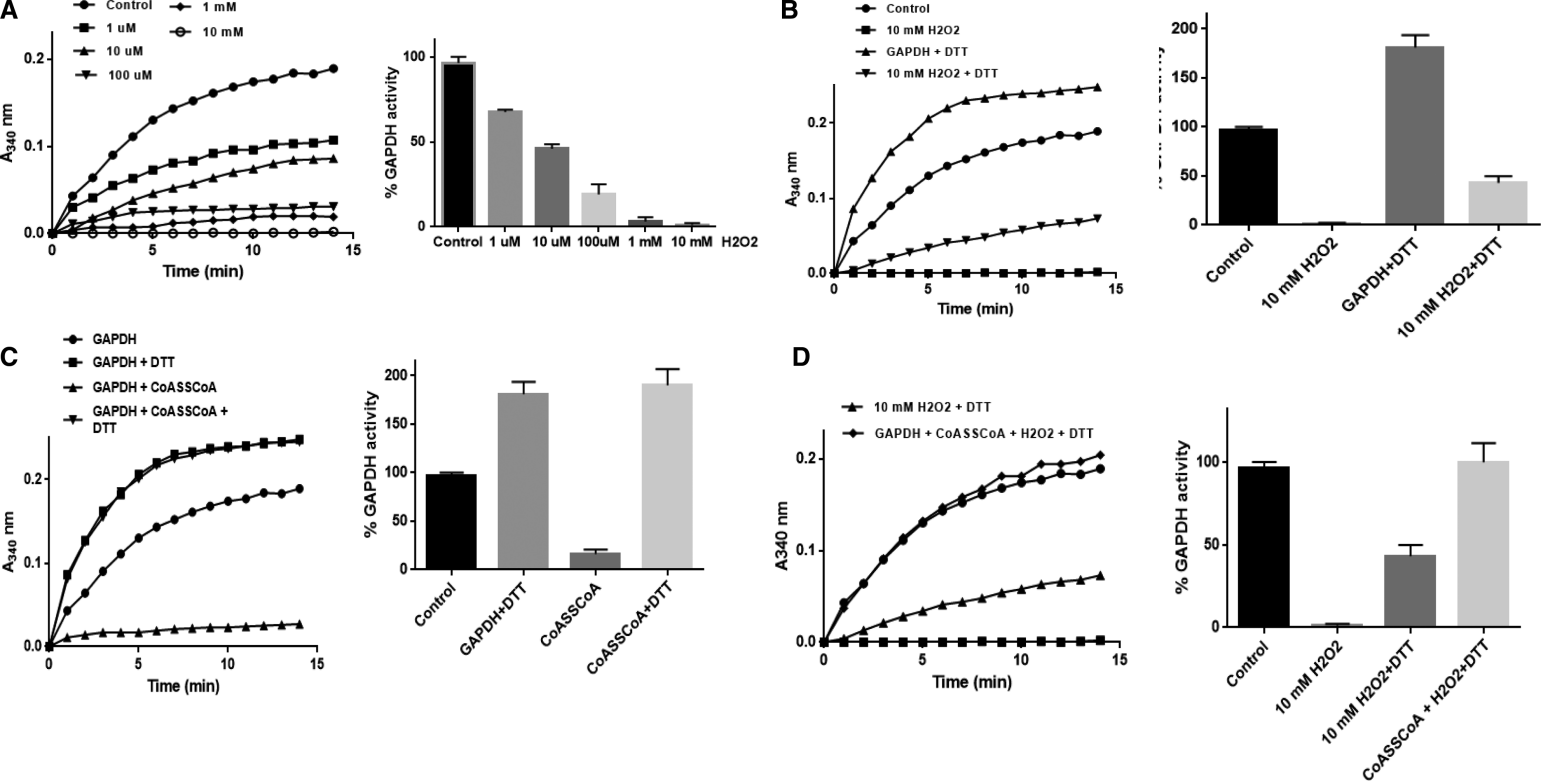
Testing the effect of CoAlation on SaGAPDH activity *in vitro*. *In vitro* CoAlation prevents H_2_O_2_-induced overoxidation of recombinant SaGAPDH dose-dependent inhibition of SaGAPDH activity by H_2_O_2_ *in vitro*. (**B**) *In vitro* CoAlation inhibits SaGAPDH activity. (**C**) H_2_O_2_-induced inhibition of SaGAPDH activity is only partially reversed by DTT. (**D**) The inhibition of SaGAPDH activity by *in vitro* CoAlation is fully reversed by DTT.

We initially examined a dose-dependent inhibition of recombinant SaGAPDH with H_2_O_2_. Purified His-SaGAPDH was incubated in the absence or presence of 1 μM, 10 μM, 100 μM, 1 mM or 10 mM H_2_O_2_ for 10 min before the activity was measured spectrophotometrically, as described in Experimental procedures. As shown in Figure 6A, exposure of SaGAPDH to 10 μM H_2_O_2_ results in a ∼50% decrease of its catalytic activity. The presence of 1 mM H_2_O_2_ in the reaction mixture resulted in 95% inhibition, while 10 mM H_2_O_2_ completely blocked SaGAPDH activity. These data indicate that SaGAPDH is efficiently inhibited by ROS-mediated direct oxidation of its catalytic active cysteine. Further analysis revealed that the inactivation of SaGAPDH activity by 10 mM H_2_O_2_ was only partially reversible, as only 40% of SaGAPDH activity could be recovered with 10 mM DTT (Figure 6B). The addition of DTT to the untreated sample of SaGAPDH increased its activity by ∼50%, indicating partial and reversible oxidation of recombinant preparations of His-SaGAPDH during the storage.

Next, we investigated the effect of CoAlation on SaGAPDH catalytic activity. As shown in Figure 6C, *in vitro* CoAlation of SaGAPDH resulted in 90% inhibition of its activity. The inhibitory effect of CoAlation on SaGAPDH activity was completely reversed by the addition of 10 mM DTT to the reaction mixture. In line with these results, we have recently reported significant inhibition (∼80%) of mammalian GAPDH by *in vitro* CoAlation, which was fully reversed by DTT [22]. These findings prompted us to examine whether CoAlation can protect SaGAPDH catalytic activity against irreversible overoxidation by H_2_O_2_. In the agreement with the above findings, SaGAPDH was fully inactivated with 10 mM H_2_O_2_ and the addition of 10 mM DTT recovered only 40% of the enzymatic activity (Figure 6D). We subsequently found that *in vitro* CoAlation of SaGAPDH before exposure to 10 mM H_2_O_2_ resulted in nearly 100% recovery of its activity with DTT. These results indicate that SaGAPDH CoAlation can prevent irreversible overoxidation of the catalytic Cys151 under the oxidative stress.

### Analysis of GAPDH/CoA interaction by MD simulation

In the glycolysis pathway, GAPDH functions to convert glyceraldehyde 3-phosphate to the energy-rich intermediate 1,3-bisphosphoglycerate and uses inorganic phosphate to harness the energy into NADH [54]. Biochemical, mutational and crystallographic studies of GAPDH from prokaryotic and eukaryotic cells have revealed that the active site cysteine is essential for the catalytic activity of the enzyme. GAPDH consists of four identical non-covalently connected subunits, which possess the NAD^+^-binding domain and the catalytic domain. Each subunit contains a strictly conserved catalytic cysteine, which is susceptible to various thiol modifications in cellular response to oxidative stress, including sulfenylation, glutathionylation, nitrosylation, bacillithiolation and mycothiolation [55]. These modifications inhibit GAPDH activity and glucose metabolism, and divert a metabolic flux through the pentose phosphate cycle to increase NADPH production.

**Figure 7.**
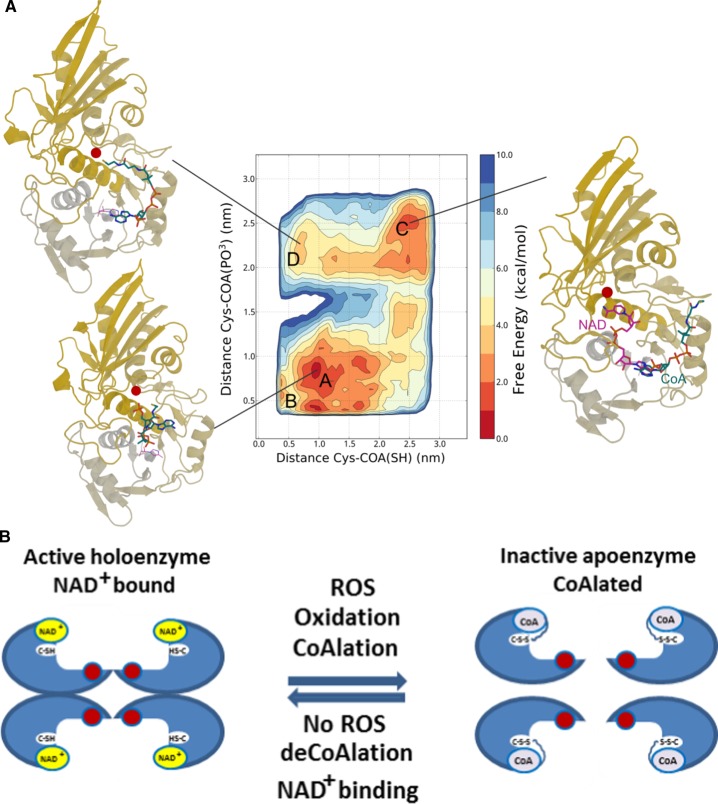
The mode of interaction between SaGAPDH and CoA. (**A**) Free energy landscape of the CoA : GAPDH complex as a function of the distance between Cys151 (shown as a red ball in the exemplary structures) and the CoA SH tail (*x*-axis) and the PO^3^ group attached to the sugar moiety (*y*-axis). The contour lines are drawn every 2 kcal/mol. CoA and, for reference, NAD are shown in blue and magenta sticks, respectively. The computed free energy landscape reveals how CoA can assume different conformations, folded onto itself (labeled as A in the map) near the nicotinamide-binding site (as shown in the corresponding figure on the lower left corner), or binding the adenine-binding site (MD-B). The CoA tail can approach Cys151 from both these minima or by assuming the conformations found in basin MD-D (top left corner) when starting from the extended conformation observed in basin MD-C (top right corner). The latter basins are slightly higher energy when compared with the absolute minimum, but still accessible. (**B**) A proposed model of CoA binding to SaGAPDH, where CoA binds to the nicotinamide-binding site and its tail approaches Cys151 forming a covalent bond.

Recently, a crystal structure of overoxidized SaGAPDH revealed an apo form of the enzyme lacking NAD^+^ in the binding pocket [25]. Taking this into account and the fact that SaGAPDH is CoAlated in response to H_2_O_2_ and NaOCl, we hypothesized that the ADP moiety of CoA may also be involved in mediating the interaction with SaGAPDH by occupying the vacant nucleotide-binding pocket. To investigate the binding mode between CoA and SaGAPDH, we performed MD simulations. In the present study, the structure of the CoA : GAPDH complex was modeled onto the GAPDH structure with NAD^+^ (PDBID: 3LVF). The adenine moieties of both cofactors were aligned to start from a structure with CoA bound in the NAD adenine-binding site. CoA remains firmly bound to the protein during a long 400 ns MD run in explicit solvent, suggesting that CoA can indeed bind to the NAD^+^ binding site. As (un)binding events can occur on a wide range of timescales, from μs to hours, they cannot be captured by the limited timescales accessible to standard MD simulations. For this reason, we employed metadynamics to enhance the sampling and accelerate the occurrence of these rare events. The free energy landscape reconstructed after a 400 ns metadynamics run is shown in Figure 7A. In this map, the most stable conformation (labeled MD-A) has CoA-folded onto itself and occupies the deep cavity in proximity of the nicotinamide-binding site. In this conformation, the CoA thiol group is quite close to catalytic Cys151 (at ∼7–8 Å, highlighted as a red sphere in the figure). A nearby secondary minimum (MD-B) is also present, structurally similar to A, but for which the CoA thiol group and Cys151 are within bond distance (SH–SH distance of 4 Å). This conformation is only 1.8 kcal/mol higher in energy, suggesting that interactions between the CoA thiol and Cys151 are not only possible, but might lead to covalent binding. An alternative metastable basin (MD-C) is also observed in the free energy map. In this conformation, CoA is bound to the NAD^+^ adenine-binding site, as in the starting X-ray structure. As in the case of MD-B, this minimum is very close in energy to lowest basin MD-A, being only 1.3 kcal/mol less stable. Interestingly, CoA can approach Cys151 also from this conformation, by assuming a bridge conformation that connects the adenine- and nicotinamide-binding sites (labeled MD-D in Figure 7A).

In the holoenzyme structure, the NAD^+^ occupies the nucleotide-binding pocket and prevents the formation of bonds with the ADP moiety of CoA. The absence of NAD^+^ in the apoenzyme structure allows this ADP moiety to occupy the nucleotide-binding pocket.

Taken together the above findings and published studies in the literature, we propose that the interaction between CoA and the oxidized form of GAPDH is facilitated by the ADP moiety of CoA which can occupy the vacant nucleotide-binding pocket, while permitting the pantetheine tail of CoA to form a covalent disulfide bond with Cys151.

## Discussion

The presence of a highly reactive thiol group and the ADP moiety in the CoA structure offer diverse functions in biochemical processes. Using a thiol group, CoA reacts with carboxylic acids to form diverse thioesters, thus functioning in cellular metabolic processes as a master acyl group carrier. It also functions as a carbonyl-activating group in numerous anabolic and catabolic processes, including the citric acid cycle and fatty acid metabolism. In addition, CoA provides the 4-phosphopantetheine prosthetic group to proteins that play key roles in fatty acid, polyketide and non-ribosomal peptide biosynthesis. A novel unconventional function of CoA in redox regulation, involving oxidative S-thiolation of cellular proteins (termed protein CoAlation), has recently emerged as a new research field [22]. In this original study, extensive protein CoAlation was observed in mammalian cells and tissues in response to oxidative and metabolic stress. Developed research tools and a mass spectrometry methodology allowed the identification of 587 CoAlated proteins under various experimental conditions in cell-based and animal models ([22] and unpublished data). Bioinformatic pathway analysis of CoAlated proteins showed that they are involved in diverse cellular processes, including metabolism, protein synthesis and stress response. Furthermore, catalytic activities of several metabolic enzymes, including creatine kinase, isocitrate dehydrogenase 2, pyruvate dehydrogenase kinase 2 and GAPDH, were shown to be inhibited by *in vitro* CoAlation. Here, we demonstrate for the first time that protein CoAlation also occurs in prokaryotic cells and is associated with redox regulation. Evidence is provided that exponentially growing Gram-negative and Gram-positive bacteria exhibit a basal level of protein CoAlation, while exposure to oxidizing agents and glucose deprivation induce strong covalent protein modification by CoA in a DTT-sensitive manner.

The intracellular concentration of CoA and its derivatives in bacteria varies from 0.4 mM in *E. coli* to low millimolar level in *S. aureus* [1,56,57]. The level of CoA and the ratio between CoA and its thioesters fluctuate depending on the growth conditions and are regulated by the availability of nutrients, intracellular metabolites and the exposure to stress. In exponentially growing *E. coli*, four CoA species (CoASH, acetyl CoA, succinyl CoA and malonyl CoA) compose the bulk of the CoA pool, where acetyl CoA is the dominant component (79.8%) and the level of CoASH is significantly lower (13.8%) [57]. When glucose in the medium is depleted, CoASH becomes the major component (82%) of the CoA pool at the expense of the acetyl CoA derivatives. The same effect was also observed in cells treated with the oxidizing agent or cultured in the glucose-deprived medium [57]. The production of metabolically active CoA thioesters has been associated with cell growth, while the increase in the level of CoASH under adverse growth conditions may allow bacteria to sense, respond and adapt to excessive ROS accumulation via thiol-mediated protein CoAlation.

The redox proteome analysis of *S. aureus* enabled us to identify 356 CoAlated proteins which belong to diverse functional classes. The main targets of CoAlation are proteins involved in cellular metabolism, translation and antioxidant response, and this pattern correlates with that in mammalian cells and tissues [22]. In contrast with mammalian CoAlome, many redox-dependent transcription factors, whose DNA-binding activity is modulated in response to cysteine oxidation, have been identified in diamide-treated *S. aureus*. These include transcriptional regulators SarR, CtsR, AgrA, PerR and SarS, which control the expression of genes involved in oxidative stress response, antibiotic resistance, virulence or catabolism of aromatic compounds.

To examine the effect of CoAlation on the activity of modified proteins, our efforts were focused on SaGAPDH, a major target of S-thiolation in prokaryotic and eukaryotic cells in response to oxidative and metabolic stress. We provide evidence that CoAlation protects the catalytic cysteine in SaGAPDH against overoxidation under H_2_O_2_ stress *in vitro* and offers a reversible mode of regeneration of this essential glycolytic enzyme during the recovery from oxidative stress. Using MD simulations to examine the binding mode between CoA and SaGAPDH, we found that in the most stable conformation, the ADP moiety of CoA occupies the deep cavity where the nicotinamide-binding pocket is located, and the CoA thiol group and Cys151 are within the distance permitting covalent disulfide bond formation. Based on these findings and a recently reported crystal structure of overoxidized SaGAPDH which lacks NAD^+^ in the binding pocket, we propose a double anchor model for GAPDH CoAlation in response to oxidative or metabolic stress. In this model, the ADP moiety of CoA anchors to the nucleotide-binding pocket in the oxidized form of GAPDH and positions the CoA thiol group in the flexible pantetheine tail in close vicinity for covalent bond formation with catalytic Cys151 (Figure 7B).

## Abbreviations

4PP: 4′-phosphopantetheine
AhpC: alkyl hydroperoxide reductase C
CoA: coenzyme A
CoASSCoA: CoA disulfide
CoASSG: CoA-cysteine and CoA-glutathione
DTT: dithiothreitol
Fbp: fructose-1,6-bisphosphate aldolase
GapA2: glyceraldehyde-3-phosphate dehydrogenase 2
GAPDH: glyceraldehyde-3-phosphate dehydrogenase
GO: Gene Ontology
GSH: glutathione
GuaB: inosine-5′-monophosphate dehydrogenase
H_2_O_2_: hydrogen peroxide
HMG: 3-hydroxy-3-methylglutaryl
HOCl: hypochlorous acid
IAM: iodoacetamide
IPTG: isopropyl-β-d-thiogalactopyranoside
LB: Luria Bertani
LMW: low-molecular-weight
MD: molecular dynamics
Mqo1/2: malate : quinone oxidoreductases 1 and 2
NaOCl: sodium hypochlorite
NB3: Nutrient Broth 3
OD_600_: optical density at 600 nm
PAGE: polyacrylamide gel electrophoresis
PpaC: manganese-dependent inorganic pyrophosphatase
ROS: reactive oxygen species
SaGAPDH: *S. aureus* glyceraldehyde-3-phosphate dehydrogenase
SDS: sodium dodecyl sulfate
TpiA: triose phosphate isomerase
Tpx: thiol peroxidase
